# In Silico Prioritization of STAT1 3′ UTR SNPs Identifies rs190542524 as a miRNA-Linked Variant with Potential Oncogenic Impact

**DOI:** 10.3390/ncrna11030032

**Published:** 2025-04-29

**Authors:** Ebtihal Kamal

**Affiliations:** Department of Basic Medical Sciences, College of Medicine, Prince Sattam bin Abdulaziz University, Al Kharj 16278, Saudi Arabia; e.kamal@psau.edu.sa

**Keywords:** 3′ UTR SNP, UTR, STAT1, miRNA, bioinformatics

## Abstract

Background: Single-nucleotide polymorphisms (SNPs) are associated with multiple disorders and various cancer types. In the context of cancer, alterations within non-coding regions, specifically 3′ untranslated regions (3′ UTR), have proven substantially important. Methods: In this study, we utilized various bioinformatics tools to examine the effect of SNPs in the 3′ UTR. We retrieved the 3′ UTR SNPs of the Signal Transducer and Activator of Transcription 1 (STAT1) gene from the National Centre for Biotechnology Information (NCBI) website. Next, we employed the Polymorphism in miRNAs and their corresponding target sites (PolymiRTS) database to predict the 3′ UTR SNPs that create new microRNA (miRNA) binding sites and their respective miRNAs. The effect of the 3′ UTR SNPs on the messenger RNA structure was studied using RNAfold server. We used Cscape tool to predict the oncogenic 3′ UTR SNPs. Then, we submitted the miRNAs to the miRNet database to visualize the miRNA-miRNAs’ target genes interaction, for which gene enrichment analysis was performed using ShinyGO. Protein–protein interactions were conducted using the STRING database. We conducted miRNA enrichment analysis utilizing miRPathDB, subsequently performing miRNA differential expression analysis through oncoMIR, and the StarBase database. The survival analysis of the upregulated miRNAs in cancer was investigated using the Kaplan–Meier Plotter. Result: Twelve SNPs were predicted to create new miRNA binding sites. Two of them, rs188557905 and rs190542524, were predicted to destabilize the mRNA structures. We predicted rs190542524, rs11305, rs186033487, and rs188557905 to be oncogenic 3′ UTR SNPs, with high-confidence predictions and scores > 0.5. Using miRNAs’ target genes enrichment analysis, this study indicated that the miRNA target genes were more likely to be involved in cancer-related pathways. Our comprehensive analysis of miRNAs, their functional enrichment, their expression in various types of cancer, and the correlation between miRNA expression and survival outcome yielded these results. Our research shows that the oncogenic 3′ UTR SNP rs190542524 creates a new binding site for the oncogenic miRNA hsa-miR-136-5p. This miRNA is significantly upregulated in BLCA, LUSC, and STAD and is linked to poor survival. Additionally, rs114360225 creates a new binding site for hsa-miR-362-3p, influencing LIHC. Conclusions: These analyses suggest that these 3′ UTR SNPs may have a functional impact on the STAT1 gene’s regulation through their predicted effect on miRNA binding sites. Future experimental validation could establish their potential role in the diagnosis and treatment of various diseases, including cancer.

## 1. Introduction

Signal Transducer and Activator of Transcription 1 (STAT1) is a dormant transcription factor in the cytoplasm that plays an important role in the interferon signaling pathway [1,2]. It belongs to a protein family comprising transcription factors crucial for regulating diverse intracellular processes, including proliferation, differentiation, apoptosis, and angiogenesis [3,4,5]. The JAK-STAT pathway, which includes STAT1, is activated in response to type I, type II interferons, and many other cytokines [2,6,7].

A wide range of diverse diseases have been linked to dysregulated STAT1 protein and defects in the JAK-STAT signaling pathway, emphasizing the importance of this pathway in maintaining cellular integrity [8,9,10].

The function and significance of STAT1 in cancer biology have been studied for a long time [11]. However, STAT1’s role in cancer biology as well as the mechanism underlying its tumor-suppressive or oncogenic properties remain unclear [11]. Most data indicate that STAT1 activation has a tumor suppressor function in cancer cells [11,12,13,14]. While other clinical studies and experimental results indicate that STAT1 has tumor-promoting properties [15,16]. Additional research indicates that STAT1 can act as both a tumor suppressor and an oncoprotein in cells with specific malignant phenotypes [11,17].

MicroRNA (miRNA) is a small, noncoding RNA with 20–22 nucleotides [18,19]. It binds to the 3′ untranslated region (3′ UTR) of messenger RNA (mRNA), inhibiting translation and ultimately resulting in mRNA degradation [20,21,22].

A single miRNA can modulate the expression of numerous mRNAs within a cell by binding to the specific 3′ UTR binding sites of the target mRNA [22]. Multiple binding sites for the same miRNA within 3′ UTRs can significantly amplify its regulatory effect [23].

Single-nucleotide polymorphisms (SNPs) are gene variations that have a higher frequency than other variations, such as insertions, deletions, and copy number variations, which can influence gene function and contribute to diversity in phenotypes and disease susceptibility [24].

SNPs modulate gene expression via various mechanisms and can be located in 5′ UTR and 3′ UTRs [25]. The 5′ UTR comprises genetic components that modulate gene expression [26]. Polymorphisms in 5′ UTRs are associated with numerous human diseases [27,28,29,30] due to their regulation of mRNA processing, transport, stability, and translation [27,31].

SNPs within miRNA binding sites in the 3′ UTR can modify miRNA–mRNA interactions and substantially affect gene expression [32,33,34].

SNPs in miRNA binding sites of proto-oncogenes may disrupt their post-transcriptional regulation, leading to overexpression of oncogenic proteins, tumor initiation, progression, and altered drug response in cancer patients [32,35,36,37]. Therefore, detecting and characterizing such mutations can improve risk assessment for healthy carriers and enhance the diagnostic and therapeutic toolbox for the patients [38].

The introduction of different bioinformatics tools and software has rendered it feasible to study and analyze SNPs in the 3′ UTRs. In this study, we used Polymorphism in miRNAs and their corresponding target sites (PolymiRTS) to predict 3′ UTR SNP-induced changes to the sequences of the miRNA targets. Since the importance of 3′ UTR SNPs has been highlighted in several studies, their association with various disorders was examined. This study aimed to investigate the effect of 3′ UTR SNPs in the *STAT1* gene using different bioinformatics methods to determine their oncogenic potential.

## 2. Results

### 2.1. Retrieval of 3′ UTR SNPs of STAT1 from NCBI

We used NCBI to retrieve the 838 SNPs in the 3′ UTR, and they were used for downstream analysis.

### 2.2. Results of the Impact of 3′ UTR SNPs on miRNA Binding Sites

We evaluated the impact of 3′ UTR SNPs on miRNA binding sites using PolymiRTS, which predicted the association between seventeen 3′ UTR SNPs and miRNAs, as well as disruption scores, conservation scores, ancestral alleles, modified alleles, context, and score modifications, as shown in Table S1. Twelve 3′ UTR SNPs created new miRNA-binding sites and their corresponding 30 miRNAs, as shown in Table 1.

### 2.3. The Effect of 3′ UTR SNPs on the Secondary Structure of mRNA

We subjected the wild-type and the twelve 3′ UTR SNPs that created new miRNA binding sites to RNAfold analysis to evaluate their potential impact on mRNA structure. RNAfold analysis revealed two out of twelve 3′ UTR SNPs (rs188557905 and rs190542524) showing MFE values rising from −35.80 kcal/mol to −13.90 kcal/mol and from −23.80 kcal/mol to −22.30 kcal/mol, respectively. They were predicted to destabilize the mRNA structure. Other SNPs have either minimal or no variation in energy, shown in Table 2.

### 2.4. Cscape Results of Cancer-Associated 3′ UTR SNPs

Cscape was used to screen the oncogenic potential of twelve 3′ UTR SNPs that created new miRNA binding sites. Rs190542524 (T/A) was predicted to be an oncogenic SNP, with the highest score of 0.802671. Meanwhile, rs11305, rs186033487, rs188557905, and rs190542524 (T/G) also had strong predictions for causing cancer, with scores above 0.7. Other SNPs, like rs139958571, rs41363648, rs41481847, rs184180073, rs79073086, and rs139958571, have lower predictions with scores between 0.5 and 0.7. Only one SNP, rs114360225, was predicted as benign, as shown in Table 3.

### 2.5. Results of miRNet Identification of miRNAs’ Target Genes

**Figure 1:**
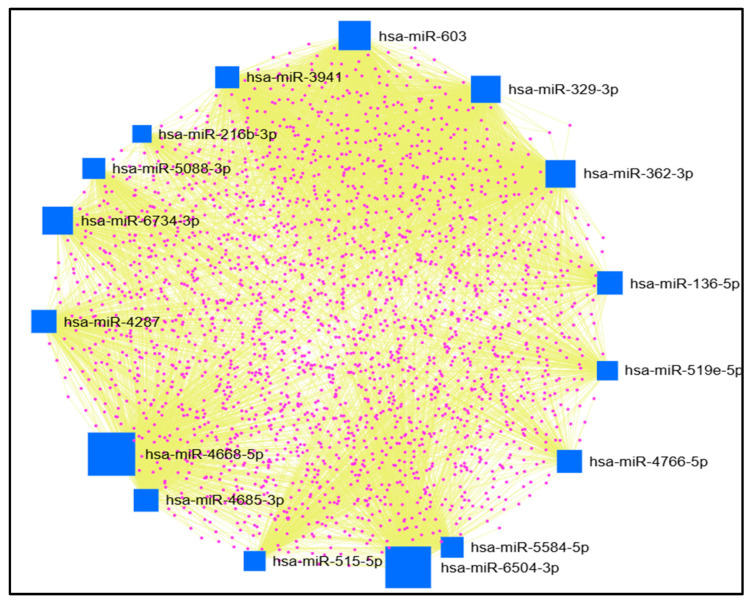
MiRNA-gene network by miRNet. Blue square nodes represent miRNAs (source nodes), purple dots represent genes (target nodes), and yellow edges represent the interactions between nodes. The size of the blue square nodes, which represent miRNAs, indicates their significance in the network. A larger square size reflects more targeted genes by that miRNA, highlighting its potential importance in gene regulation.

We submitted the 30 miRNAs that bind to the new miRNA binding sites created by 3′ UTR SNPs to the miRNet. Only 16 miRNAs (hsa-miR-329-3p, hsa-miR-603, hsa-miR-3941, hsa-miR-4668-5p, hsa-miR-6504-3p, hsa-miR-4287, hsa-miR-4685-3p, hsa-miR-6734-3p, hsa-miR-515-5p, hsa-miR-519e-5p, hsa-miR-5088-3p, hsa-miR-136-5p, hsa-miR-5584-5p, hsa-miR-4766-5p, hsa-miR-216b-3p, and hsa-miR-362-3p) had ≥100 interactions and underwent further analysis. MiRNet predicted 2384 genes interacting with the 16 miRNAs, as presented in Figure 1.

### 2.6. Result of Gene Enrichment Analysis

**Figure 2:**
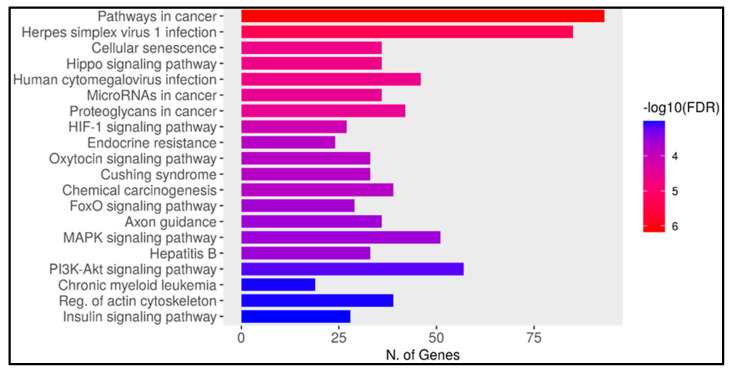
Bar plot of gene enrichment pathways by ShinyGo. The *Y*-axis represents the top 20 significant pathways. The *X*-axis represents the number of genes in the pathway.

We used ShinyGo to study the gene enrichment pathways of the miRNAs’ target genes. We found that 92 genes were significantly enriched in the pathway of cancer (*p*-value 2.04 × 10^−5^). Fifty genes in the MAPK signaling pathway (*p*-value 0.0023), forty-one genes in proteoglycans in cancer (*p*-value 0.00025), thirty-seven genes in the microRNAs in cancer pathway (*p*-value 8.97 × 10^−5^), and twenty-eight genes were enriched in the FoxO signaling pathway (*p*-value 0.0011), as presented in Figure 2. The complete enrichment table is presented in Table S2.

### 2.7. Protein–Protein Interaction and Disease–Gene Association Enrichment

**Figure 3:**
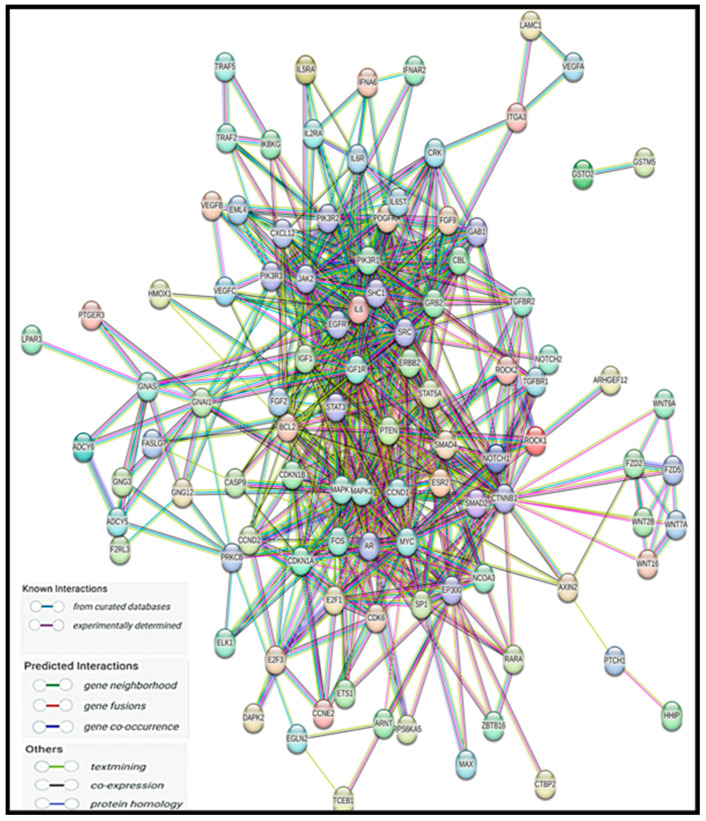
Protein–protein interaction network using STRING database.

We submitted the 2384 miRNAs’ target genes into multiple protein modules of STRING. Protein–protein interaction is illustrated in Figure 3. Next, we used STRING to understand the association between diseases and miRNAs’ target genes through disease–gene association enrichment analysis. The majority of genes involved in pathways of cancer, organ system cancer, gastrointestinal cancer, and hepatobiliary system cancer are shown in Figure S1.

### 2.8. Ten miRNAs Enriched in the Pathway of Cancer

We used miRPathDB to construct a heat map of miRNA enrichment. We found that ten out of the sixteen miRNAs were enriched in the pathways in cancer, namely hsa-miR-3941 (−log *p*-value > 2.220), hsa-miR-4668-5p (−log *p*-value > 2.626), hsa-miR-4287 (−log *p*-value > 2.679), hsa-miR-4685-3p (−log *p*-value > 2.469), hsa-miR-6734-3p (−log *p*-value > 4.891), hsa-miR-515-5p (−log *p*-value > 1.375), hsa-miR-519e-5p (−log *p*-value > 1.302), hsa-miR-5088-3p (−log *p*-value > 2.303), hsa-miR-136-5p (−log *p*-value > 1.524), and hsa-miR-5584-5p (−log *p*-value > 3.697).

**Figure 4:**
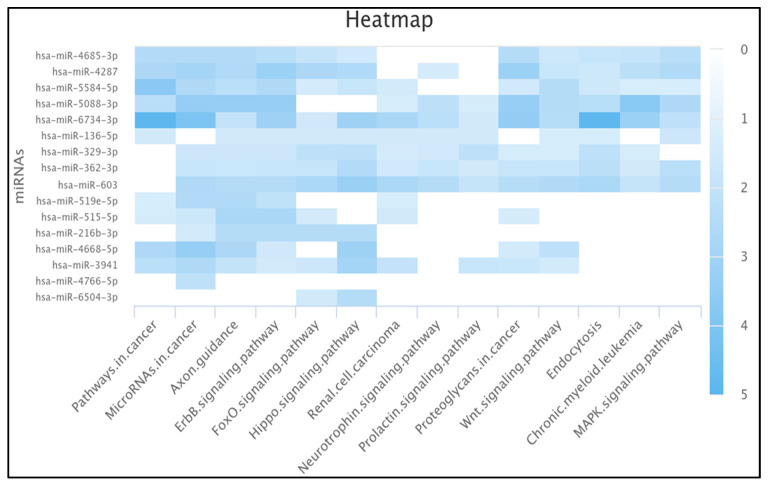
The heat map shows the miRNA enrichment constructed by miRPathDB. Darker colors indicate stronger statistical significance (higher log *p*-values and lower *p*-values), while lighter colors suggest weaker associations (lower log *p*-values and higher *p*-values).

Moreover, we found 8 out of 16 miRNAs (hsa-miR-603, hsa-miR-4287, hsa-miR-4685-3p, hsa-miR-6734-3p, hsa-miR-5088-3p, hsa-miR-136-5p, hsa-miR-5584-5p, and hsa-miR-362-3p) were enriched in the MAPK signaling pathway, 11 miRNAs (hsa-miR-329-3p, hsa-miR-603, hsa-miR-3941, hsa-miR-4668-5p, hsa-miR-4287, hsa-miR-4685-3p, hsa-miR-6734-3p, hsa-miR-515-5p, hsa-miR-5088-3p, hsa-miR-5584-5p, and hsa-miR-362-3p) in proteoglycans in cancer, 14 miRNAs (hsa-miR-329-3p, hsa-miR-603, hsa-miR-3941, hsa-miR-4668-5p, hsa-miR-4287, hsa-miR-4685-3p, hsa-miR-6734-3p, hsa-miR-515-5p, hsa-miR-519e-5p, hsa-miR-5088-3p, hsa-miR-5584-5p, hsa-miR-4766-5p, hsa-miR-216b-3p, and hsa-miR-362-3p) in microRNAs in cancer, and 12 (hsa-miR-329-3p, hsa-miR-603, hsa-miR-3941, hsa-miR-6504-3p, hsa-miR-4287, hsa-miR-4685-3p, hsa-miR-515-5p, hsa-miR-5088-3p, hsa-miR-136-5p, hsa-miR-5584-5p, hsa-miR-216b-3p, and hsa-miR-362-3p) in the FoxO signaling pathway. Complete miRNA enrichment is shown in Figure 4.

### 2.9. Results of miRNA Differential Expression Analysis in Human Cancer

We used oncoMIR to predict the significance of miRNA differential expression across cancer types. We found that only five miRNAs (hsa-miR-362-3p, hsa-miR-136-5p, hsa-miR-515-5p, hsa-miR-515-5p, and hsa-miR-329-3p) had significant differential expression in cancer.

Hsa-miR-362-3p exhibited upregulation in bladder carcinoma (BLCA), breast invasive carcinoma (BRCA), liver hepatocellular carcinoma (LIHC), stomach adenocarcinoma (STAD), and uterine corpus endometrial carcinoma (UCEC). Moreover, hsa-miR-136-5p showed upregulation in five cancer types: BLCA, BRCA, lung adenocarcinoma (LUAD), lung squamous cell carcinoma (LUSC), and STAD (*p*-value 2.14 × 10^−2^). Additionally, hsa-miR-329-3p was upregulated in STAD (*p*-value 4.45 × 10^−3^). Furthermore, hsa-miR-515-5p was upregulated in normal lung tissue compared to LUAD, as shown in Table 4.

We used the StarBase database to visualize the differential expression of four significant differentially expressed miRNAs predicted by oncoMIR (hsa-miR-362-3p, hsa-miR-136-5p, hsa-miR-329-3p, and hsa-miR-515-5p). The results are presented as a box plot in Figure S2.

### 2.10. Survival Analysis Study of the Significantly UpRegulated miRNAs in Cancer

We used the Kaplan–Meier plotter to study the survival analysis of the significantly upregulated miRNAs in cancer tissues by both oncoMIR and StarBase. We found that the upregulated group of hsa-miR-362-3p was significantly associated with poor survival in LIHC (Logrank *p* = 0.0046), while the upregulated group of hsa-miR-136-5p was significantly associated with poor survival in BLCA (Logrank *p* = 0.038), LUSC (Logrank *p* = 0.021), and STAD (Logrank *p* = 0.00053), as shown in Figure S3

**Figure 5:**
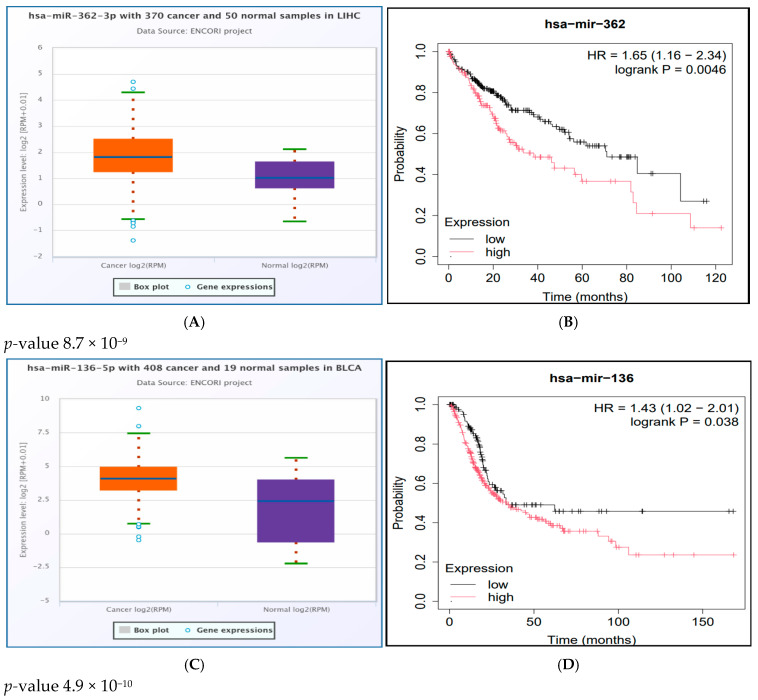

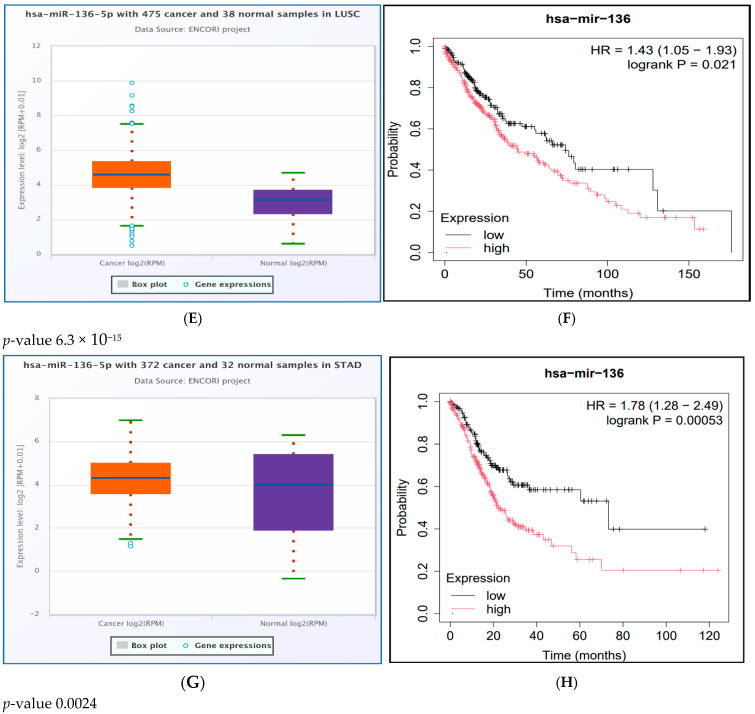
Integration of expression analysis (from the StarBase database) and survival study (from the Kaplan–Meier Plotter) of hsa-miR-362-3p and hsa-miR-136-5p. Expression and survival analysis of hsa-miR-362-3p in LIHC (**A**,**B**). Expression and survival analysis of hsa-miR-136-5p in BLCA (**C**,**D**), LUSC (**E**,**F**), and STAD (**G**,**H**).

**Figure 6:**
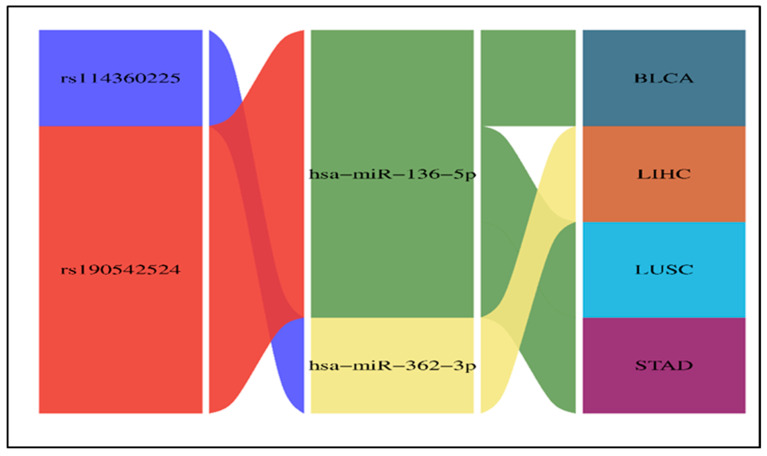
The Sankey diagram illustrates the relationships between SNPs (rs114360225 and rs190542524), the potential oncogenic miRNAs (hsa-miR-362-3p and hsa-miR-136-5p), and their corresponding cancer types.

By integration of expression analysis and survival study, we found that hsa-miR-362-3p was significantly upregulated and associated with poor survival in LIHC. Additionally, hsa-miR-136-5p was significantly upregulated and associated with poor survival in BLCA, LUSC, and STAD, as shown in Figure 5. Next, the interaction between the 3′ UTR SNP, miRNA, and the target tumor is visualized by the Sankey diagram in Figure 6.

## 3. Materials and Methods

**Figure 7:**
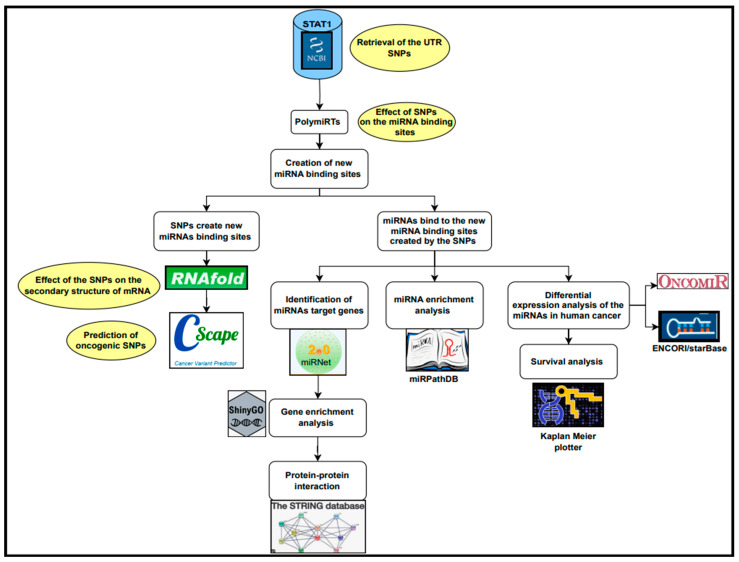
Workflow of the study.

The methods of this study are summarized in Figure 7.

### 3.1. Retrieval of 3′ UTR SNPs

We collected data for the human *STAT1* gene from the National Centre for Biotechnology Information (NCBI) website (available at https://www.ncbi.nlm.nih.gov/). We retrieved the 3′ UTR SNPs from NCBI on 20 December 2024 and analyzed them with the bioinformatics tools.

### 3.2. Evaluation of the Impact of 3′ UTR SNPs on miRNA Binding Sites

We evaluated the 3′ UTR SNPs for their correlation with miRNA binding sites using (PolymiRTS) database (accessible at https://compbio.uthsc.edu/miRSNP/ (accessed on 21 December 2024), which predicts the impact of 3′ UTR SNPs in miRNA target sites; whether they disrupt a conserved (D), non-conserved (N), or create a new miRNA binding site that could lead to abnormal gene repression (C); or in other cases when the ancestral allele cannot be determined (O). The PolymiRTS database calculates the differences in context + scores between the reference and derived alleles for each 3′ UTR SNP in the miRNA target sites.

A difference with a more negative value of the context + score indicates an increased likelihood that the polymorphism significantly altered miRNA targeting of the sequence [39]. This study was conducted on 3′ UTR SNPs that create new miRNA binding sites and were subjected to further analysis.

### 3.3. Determination of the Effect of SNPs on the Secondary Structure of mRNA

We utilized the RNAfold server from the Vienna RNA package (available at http://rna.tbi.univie.ac.at/cgi-bin/RNAWebSuite/RNAfold.cgi (accessed on 22 December 2024)) to examine how 3′ UTR SNPs that create new miRNA binding sites affect the mRNA’s secondary structure [40]. The input is the wild-type and SNP sequences that were obtained from NCBI. We obtained the mRNA secondary structures and minimum free energies (MFE) of both the wild-type mRNA and 3′ UTR SNPs. This package is based on MFE algorithms.

### 3.4. Prediction of Cancer-Associated SNPs

We used the CScape tools (available at http://cscape.biocompute.org.uk/ (accessed on 22 December 2024)) to predict the relationship between 3′ UTR SNP and cancer development. CScape is a combinatorial tool that predicts if a single mutation in a coding or noncoding part of the genome is oncogenic. The format for the input data was as follows: chromosome, position, reference, and mutant [41]. 3′ UTR SNPs are classified as neutral or oncogenic based on *p*-values ranging from 0 to 1. The tool predicts that a *p*-value >0.5 will be oncogenic, while *p*-value < 0.5 is considered neutral.

### 3.5. Identification of miRNAs’ Target Genes

MiRNet (available at https://www.mirnet.ca/ (accessed on 25 December 2024)) is a web-based platform designed to analyze and visualize miRNA regulatory networks. It integrates various datasets, including miRNA–target gene interactions, pathways, and expression profiles, enabling comprehensive analyses of miRNA functions [42]. We used the miRNet web interface to retrieve the miRNAs’ target genes and visualize the miRNA–gene interaction network. In this study, we used a degree filter for all miRNA nodes; each miRNA with a degree ≥ 100 was included.

### 3.6. Gene Enrichment Analysis

ShinyGo version (V 0.82) (available at https://bioinformatics.sdstate.edu/go/ (accessed on 27 December 2024)) is a graphical web application designed for gene-set enrichment analysis; the input is miRNAs’ target genes predicted by miRNet. It obtains insights into enriched Gene Ontology (GO) terms and pathways, facilitating the interpretation of biological data [43]. We used ShinyGO to analyze miRNAs’ target genes enrichment and visualize the enriched GO terms and pathways.

### 3.7. Protein–Protein Interaction Using the STRING Database

STRING (available at https://string-db.org/ (accessed on 21 December 2024)) is a biological database that provides a comprehensive resource for known and predicted protein–protein interactions. We submitted the miRNAs’ target genes into multiple protein modules of the STRING database. The threshold for visualizing the protein–protein interaction network was set at a medium confidence score of ≥0.4.

The STRING database integrates various types of associations, including direct physical interactions and indirect functional relationships among proteins [44]. STRING also includes tools for functional enrichment analysis, which investigates disease–gene associations that are overrepresented in a set of proteins.

### 3.8. MiRNAs Enrichment Analysis

MiRNA enrichment analysis aims to identify and understand the biological significance of miRNAs. MiRPathDB (available at https://mpd.bioinf.uni-sb.de/ (accessed on 28 December 2024)) is a comprehensive database that provides information on miRNAs, their target genes, and their biological pathways [45]. The input is miRNA ID; the output is a heat map visualizing miRNA-pathway enrichment, with the −log *p*-value representing the significance. The transformation of the *p*-value to −log *p*-value is performed to convert the *p*-value into a positive scale, making it easier to visualize and interpret in graphical representations like heat maps.

### 3.9. Pan-Cancer miRNA Differential Expression Analysis

OncoMIR (https://oncomir.org/oncomir/search_miR_tumor.html (accessed on 5 January 2025) is an online resource designed to explore miRNA expression in cancer [46]. The input is a full miRNA name to retrieve a list of cancer types where tumor formation is closely associated with the expression of the selected miRNA. Significance is determined by the Student’s paired *t*-test, comparing the difference in expression between normal and tumor tissues obtained from patients.

StarBase (available at https://rnasysu.com/encori/ (accessed on 6 January 2025)) is a comprehensive resource for exploring miRNA-target gene interaction maps. It is a biological database that focuses on decoding different RNA interactions, such as those between miRNA and mRNA, miRNA and long noncoding RNA, and protein and noncoding RNA [47]. We used this resource to create a box plot to show the differential expression of miRNAs in various cancer types. For a significant result, *p*-value < 0.05.

### 3.10. MiRNAs Survival Analysis

The Kaplan–Meier plotter (available at https://kmplot.com/analysis/ (accessed on 8 January 2025)) is a tool used to assess the correlation between gene expression (including mRNA, miRNA, proteins, and DNA) and survival outcomes in large datasets, with over 35,000 samples available for analysis [48]. It is commonly utilized in cancer research to identify biomarkers with prognostic significance.

SR plot (available at https://www.bioinformatics.com.cn/en (accessed on 12 January 2025)) is a freely accessible, easy-to-use web server that integrates all of the commonly used data visualization and graphing functions [49]. We used the SR plot to create a Sankey diagram, which is used to visualize the relation between miRNAs, associated 3′ UTR SNPs, and cancer.

## 4. Discussion

In the current study, a total of 838 SNPs in the 3′ UTR were retrieved from NCBI.

We used the PolymiRTS tool to study the effect of the 3′ UTR SNPs on the miRNA binding sites. Twelve 3′ UTR SNPs were predicted to create thirty new miRNA binding sites (Table 1). These SNPs were Rs11305, rs184180073, rs41363648, rs188557905, rs186033487, rs79073086, rs114360225, rs139958571, rs182394503, rs190542524, rs182725919, and rs41481847. Next, we used the RNAfold server to predict how the twelve 3′ UTR SNPs would affect the secondary structure of the mRNA. Only two 3′ UTR SNPs (rs188557905 and rs190542524) were predicted to destabilize the secondary structure of mRNAs, which may result in lower levels of mRNA (Table 2). Previous studies correlated with the findings that 3′ UTR SNPs have a destabilizing effect on the mRNA with many diseases [50,51]. A study comparing two groups found that a specific 3′ UTR SNP, rs929271, on the leukemia inhibitory factor (LIF) destabilized the secondary structure of mRNA. In a different study, the rs2229611 SNP in the G6PC1 gene was found to decrease the stability of mRNA using RNAfold, which was linked to a more severe disease phenotype in GSD-Ia patients [52]

To examine how 3′ UTR SNPs might cause cancer, we used the Cscape tool and found eleven out of the twelve 3′ UTR SNPs were predicted to be oncogenic (score > 0.5), with rs190542524 (T/A) having the highest confidence oncogenic prediction (score 0.802671). One SNP, rs114360225, was predicted as benign, with a low-confidence neutral prediction (score 0.468060), as shown in Table 3.

ClinVar classifies all 3′ UTR SNPs predicted as oncogenic in Cscape as benign, except rs186033487 and rs188557905, which have classification of uncertain significance.

Next, we used miRNet to find the miRNAs’ target genes (Figure 1). Next, we utilized ShinyGO and miRPathDB to conduct gene and miRNA enrichment analyses to elucidate the pathways associated with miRNAs’ target genes and miRNAs, respectively.

We found that both miRNAs’ target genes and miRNAs were significantly enriched in the same pathways involved in tumorigenesis, angiogenesis, and cell proliferation; the pathways were pathways in cancer, the MAPK signaling pathway, proteoglycans in cancer, microRNAs in the cancer pathway, and the FoxO signaling pathway (Figure 2). We found that 92 miRNA target genes were significantly enriched in the pathways in cancer (*p*-value 2.04 × 10^−5^), and 50 genes were enriched in the MAPK signaling pathway (*p*-value 0.0023). Research has shown that the MAPK pathway influences cell proliferation, survival, and differentiation and thus plays a critical role in cancer biology [53]. The gene enrichment analysis revealed that 41 genes were enriched in proteoglycans in cancer (*p*-value 0.00025) and 37 genes in the microRNAs in the cancer pathway (*p*-value 8.97 × 10^−5^). Additionally, 28 genes were enriched in the FoxO signaling pathway (*p*-value 0.0011), which is involved in transcription factors that regulate various cellular processes such as apoptosis, cell cycle control, and metabolism and is frequently implicated in cancer biology (Table S2).

Moreover, MiRNAs play a crucial and multifaceted role in cancer pathways. They act as both oncogenes and tumor suppressor genes [54]. They can also influence cellular processes such as cell cycle regulation, apoptosis, angiogenesis, and metastasis [55,56]

Our miRNA enrichment result showed 10 out of 16 miRNAs were enriched in the pathways in cancer, namely hsa-miR-3941, hsa-miR-4668-5p, hsa-miR-4287, hsa-miR-4685-3p, hsa-miR-6734-3p, hsa-miR-515-5p, hsa-miR-519e-5p, hsa-miR-5088-3p, hsa-miR-136-5p, and hsa-miR-5584-5p. Moreover, we found 8 out of 16 miRNAs were enriched in the MAPK signaling pathway, 11 in proteoglycans in cancer, 14 in microRNAs in cancer, and 12 in the FoxO signaling pathway (Figure 4).

Differential expression analysis of miRNAs in cancer from the oncoMIR and StarBase databases showed that hsa-miR-362-3p was upregulated in BLCA, BRCA, LIHC, STAD, and UCEC; hsa-miR-136-5p was upregulated in BLCA, LUAD, LUSC, and STAD; the StarBase database predicted hsa-miR-136-5p to be insignificantly upregulated in BRCA (*p*-value 0.82), while oncoMIR predicted it as significantly upregulated (*p*-value 6.39 × 10^−4^), and hsa-miR-329-3p was upregulated in STAD. Both oncoMIR and StarBase databases predicted hsa-miR-515-5p as significantly downregulated in LUAD, with *p*-values of 4.89 × 10^−2^ and 0.0003, respectively).

Upon reviewing the literature, we found that only hsa-miR-515-5p has some relevance in the context of the STAT1 gene, particularly in thyroid cancer, where miR-515-5p was downregulated [57]. However, a direct association between hsa-miR-515-5p and STAT1 is not explicitly detailed in the literature, suggesting further exploration may be needed.

Hypothetically, oncogenic miRNAs should be upregulated in the tumor tissue and associated with poor survival. We integrated the results of expression analysis by the StarBase database and survival analysis by the Kaplan–Meier Plotter. We found that hsa-miR-362-3p was significantly upregulated and linked to poor survival in LIHC. On the other hand, hsa-miR-136-5p was significantly upregulated and linked to poor survival in BLCA, LUSC, and STAD (Figure 5). As a result, both hsa-miR-362-3p and hsa-miR-136-5p may have a potential oncogenic role in cancer. They were predicted to recognize new miRNA binding sites created by rs114360225 (predicted as a benign SNP by Cscape) and rs190542524 (predicted as an oncogenic SNP by Cscape), respectively.

The effect of rs114360225 and rs190542524 on cancer is not directly addressed in the literature. However, both genetic variants can influence cancer susceptibility and progression by regulating gene expression and signaling pathways. Alteration of the STAT1 expression can affect the cellular response to stress and inflammation, potentially influencing cancer development. Both 3′ UTR SNPs may interfere with STAT1 regulation by creating new miRNA binding sites that downregulate its expression, potentially impairing its tumor suppressor effect. While STAT1 protein is involved in various signaling pathways that regulate cell growth and apoptosis. Genetic variants may affect these pathways and result in an increase in cancer cell proliferation or resistance to apoptosis.

In agreement with our result, which is presented in Figure S2Q, the expression of hsa-miR-136-5p was studied in a cohort of 1242 samples provided by the Gene Expression Omnibus and the Cancer Genome Atlas. They found that hsa-miR-136-5p is upregulated in LUAD versus normal tissues and involved in the molecular mechanism of LUAD through inhibiting the expressions of downstream genes involved in cell adhesion (claudin-18, sialophorin, and syndecan 2) [58]. However, we found that its upregulation is non-significantly associated with poor survival (*p*-value 0.07), as shown in Figure S3G. As part of a different study, a genome-wide miRNA microarray was used to find miRNAs that were differentially expressed in hepatocellular carcinoma on cirrhotic livers. They found hsa-miR-136 was downregulated [59]. This result is in agreement with ours: hsa-miR-136 is significantly downregulated in LIHC (*p*-value 2.1 × 10^−5^), as presented in Figure S2P. Several types of cancer have been linked hsa-miR-362-3p. Studies indicate its association with breast [60,61], colorectal [62], cervical [63,64], and ovarian cancer [61,65,66,67], suggesting it may influence cancer progression and patient prognosis.

To our knowledge, there is no published study correlating hsa-miR-136-5p to BLCA, LUSC, or STAD. Additionally, there is no published information discussing the expression of hsa-miR-362-3p in LIHC.

## 5. Conclusions

We concluded that the oncogenic 3′ UTR SNP rs190542524 was predicted to create a new binding site for the oncogenic miRNA hsa-miR-136-5p, which is significantly upregulated in BLCA, LUSC, and STAD and associated with poor survival. Additionally, rs114360225 was predicted to create a new binding site for hsa-miR-362-3p, influencing LIHC and associated with poor survival (Figure 6).

However, there are some limitations to the current study, such as the bioinformatics analysis performed on 3′ UTR variants of STAT1 not being disease-specific. The functional significance of the UTR variants determined via bioinformatics analysis requires experimental validation. Future functional studies could provide insights into the role of STAT1 in cancer biology by investigating how rs190542524 and rs114360225 affect its function at the molecular level. Understanding the impact of the 3′ UTR SNPs on cancer could lead to personalized treatment strategies, particularly in tailoring immunotherapies that leverage the immune system’s response to tumors.

## Figures and Tables

**Table 1:** 3′ UTR SNPs created new miRNA-binding sites and their corresponding miRNAs.

SNP ID	miRNAs
rs11305	hsa-miR-6504-3p
rs184180073	hsa-miR-4287, hsa-miR-4685-3p, hsa-miR-5088-3p, hsa-miR-6734-3p
rs41363648	hsa-miR-134-3p, hsa-miR-4778-5p, hsa-miR-7114-5p
rs188557905	hsa-miR-4699-3p
rs186033487	hsa-miR-122-3p
rs79073086	hsa-miR-4766-5p
rs114360225	hsa-miR-216b-3p, hsa-miR-329-3p, hsa-miR-362-3p, hsa-miR-3941, hsa-miR-603
rs139958571	hsa-miR-6814-3p, hsa-miR-6872-5p
rs182394503	hsa-miR-202-5p, hsa-miR-337-3p
rs190542524	hsa-miR-136-5p, hsa-miR-515-5p, hsa-miR-519e-5p
rs182725919	hsa-miR-6741-5p
rs41481847	hsa-miR-3148, hsa-miR-3162-5p, hsa-miR-4668-5p, hsa-miR-5584-5p, hsa-miR-6750-5p, hsa-miR-6822-5p

**Table 2:** Comparison between the wild-type and mutant mRNA structures of the predicted 3′ UTR SNPs that create new miRNA binding sites using the RNAfold server.

SNP ID	Minimum Free Energy of Wild Type kcal/mol	Wild mRNA	Minimum Free Energy of Mutant Type kcal/mol	Mutant mRNA	Interpretation
rs114360225	−16.70	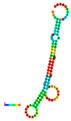	−17.20	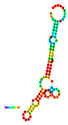	A reduction in MFE in the mutant mRNA induces structural alterations in the mRNA, hence stabilizing its structure.
rs139958571	−10.40	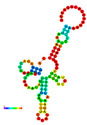	−10.40	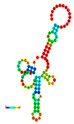	No alteration in energy, accompanied by no alteration in mRNA structure.
rs41363648	−10.30	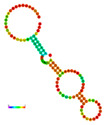	−10.30	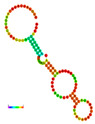	No alteration in energy, accompanied by no alteration in mRNA structure.
rs41481847	−21.40	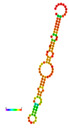	−26.00	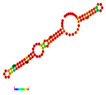	A reduction in MFE in the mutant mRNA induces structural alterations in the mRNA, hence stabilizing its structure.
rs11305	−19.40	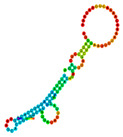	−19.40	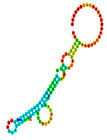	No alteration in energy, accompanied by no alteration in mRNA structure.
rs184180073	−10.40	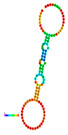	−10.40	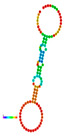	No alteration in energy, accompanied by no alteration in mRNA structure.
rs79073086	−11.50	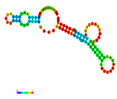	−12.50	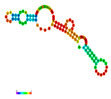	A reduction in MFE in the mutant mRNA induces structural alterations in the mRNA, hence stabilizing its structure.
rs41363648	−10.30	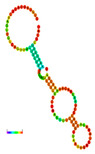	−10.30	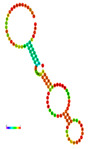	No alteration in energy, accompanied by no alteration in mRNA structure.
rs186033487	−10.70	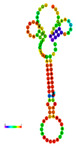	−10.90	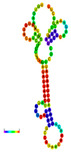	A reduction in MFE in the mutant mRNA induces structural alterations in the mRNA, hence stabilizing its structure.
rs188557905	−35.80	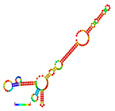	−13.90	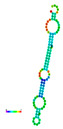	The energy elevation destabilizes the mRNA structure.
rs139958571	−10.40	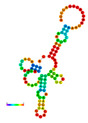	−10.40	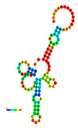	No alteration in energy, accompanied by no alteration in mRNA structure.
rs190542524	−23.80	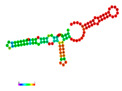	−22.30	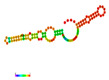	The energy elevation destabilizes the mRNA structure.

Structures are color-annotated to indicate base-pairing probabilities. The highest probabilities are red (≥99%), orange (99% > probability ≥ 95%), yellow (95% > probability ≥ 90%), dark green (90% > probability ≥ 80%), light green (80% > probability ≥ 70%), light blue (70% > probability ≥ 60%), dark blue (60% > probability ≥ 50%), and purple (≤50%).

**Table 3:** The predicted oncogenic 3′ UTR SNPs of the STAT1 gene by CScape.

SNP ID	Chromosomal Location	Cscape Score	Interpretation
rs114360225	2,190970130,T,C	0.468060	Benign
rs139958571	2,191835001,C,G	0.589398	Oncogenic
rs41363648	2,191834487,T,C	0.686973	Oncogenic
rs41481847	2,191835275,A,G	0.527544	Oncogenic
rs11305	2,191834030,T,A 2,191834030,T,C	0.707913 0.516490	Oncogenic Oncogenic
rs184180073	2,191834477,T,C	0.667051	Oncogenic
rs79073086	2,191834832,G,A 2,191834832,G,C	0.666260 0.596381	Oncogenic Oncogenic
rs41363648	2,191834487,T,C	0.686973	Oncogenic
rs186033487	2,191834759,A,C 2,191834759,A,G	0.717679 0.672660	Oncogenic Oncogenic
rs188557905	2,191834574,C,T	0.753580	Oncogenic
rs139958571	2,191835001,C,G	0.589398	Oncogenic
rs190542524	2,191835125,T,A 2,191835125,T,C 2,191835125,T,G	0.802671 0.534609 0.746269	Oncogenic Oncogenic Oncogenic

**Table 4:** Differential expression profile of miRNAs in cancer by oncoMIR.

miRNAs	Cancer Type	*p*-Value	Upregulated in
hsa-miR-362-3p	BLCA	6.86 × 10^−3^	Tumor
	BRCA	3.61 × 10^−3^	Tumor
	HNSC	3.07 × 10^−6^	Normal
	KIRC	4.77 × 10^−8^	Normal
	KIRP	2.08 × 10^−5^	Normal
	LIHC	7.37 × 10^−3^	Tumor
	LUSC	4.55 × 10^−7^	Normal
	STAD	2.12 × 10^−7^	Tumor
	THCA	1.17 × 10^−5^	Normal
	UCEC	2.49 × 10^−2^	Tumor
hsa-miR-136-5p	BLCA	4.01 × 10^−2^	Tumor
	BRCA	6.39 × 10^−4^	Tumor
	HNSC	3.36 × 10^−6^	Normal
	KIRC	1.51 × 10^−14^	Normal
	KIRP	1.09× 10^−11^	Normal
	LIHC	2.47× 10^−11^	Normal
	LUAD	4.05 × 10^−6^	Tumor
	LUSC	2.51 × 10^−6^	Tumor
	STAD	2.14 × 10^−2^	Tumor
	THCA	6.54 × 10^−7^	Normal
hsa-miR-515-5p	LUAD	4.89 × 10^−2^	Normal
hsa-miR-329-3p	BRCA	7.22 × 10^−9^	Normal
	HNSC	2.42 × 10^−3^	Normal
	STAD	4.45 × 10^−3^	Tumor

BLCA: bladder carcinoma; BRCA: breast invasive carcinoma; HNSC: head and neck squamous cell carcinoma; KIRC: kidney renal clear cell carcinoma; KIRP: kidney renal papillary cell carcinoma; LIHC: liver hepatocellular carcinoma; LUSC: lung squamous cell carcinoma; STAD: stomach adenocarcinoma; THCA: thyroid carcinoma; UCEC: uterine corpus endometrial carcinoma.

## Data Availability

The original contributions presented in this study are included in the article/Supplementary Materials. Further inquiries can be directed to the corresponding author.

## Supplementary Materials

The following supporting information can be downloaded at https://www.mdpi.com/article/10.3390/ncrna11030032/s1. Table S1: PolymiRTS analysis showing chromosomal location, ancestral alleles, allele, miRNA ID, conservation score, miRNA binding site and functional classification of the SNPs in the 3′ UTR of STAT1 gene; Table S2: miRNA target genes enrichment analysis by ShinyGo; Figure S1: Disease-gene association enrichments miRNAs’ target genes using STRING database; Figure S2: The expression analysis of the four significant differentially expressed miRNAs (hsa-miR-362-3p (A–J), hsa-miR-136-5p (K–T), hsa-miR-329-3p (T–W)) and hsa-miR-515-5p (X), in human cancer using StarBase database; Figure S3: Survival analysis of the significantly up-regulated miRNAs in cancer (hsa-miR-362 (A–D), hsa-miR-136 (E–I), using Kaplan Meier Plotter.
