# Supplementary material for: In Silico Prioritization of STAT1 3′ UTR SNPs Identifies rs190542524 as a miRNA-Linked Variant with Potential Oncogenic Impact
Source: Noncoding RNA. 2025 Apr 29;11(3):32. doi: 10.3390/ncrna11030032 (PMC12101234; doi:10.3390/ncrna11030032)
Supplement: Supplementary file 1 [file ncrna-11-00032-s001.zip › ncrna-3543927-supplementary.pdf]

Table S1. PolymiRTS analysis showing chromosomal location, ancestral alleles, allele, miRNA ID, conservation score, miRNA binding site and functional classification of the SNPs in the 3′ UTR of STAT1 gene.

Chromosomal Location	Location of the SNPs in relation to the major transcript (ENST00000361099.8)	dbSNP ID	Ancestral Allele	Allele	miR ID	Conservation	MiRSite	Functional Class	context+ score change
2:190969304	ENST00000361099.8:c.*1399A>T	rs11305	A	A	hsa-miR-656-3p	8	ttaaatATAATAT	D	-0.029
				G	hsa-miR-6504-3p	7	ttaaaTGTAATAt	C	-0.003
2:190969751	ENST00000361099.8:c.*952A>G	rs184180073	A						
				G	hsa-miR-4287	3	tgggAAGGGAGta	C	-0.09
					hsa-miR-4685-3p	3	tgggAAGGGAGta	C	-0.109
					hsa-miR-5088-3p	3	tggGAAGGGAgta	C	-0.084
					hsa-miR-6734-3p	2	tGGGAAGGgagta	C	-0.183
2:190969761	ENST00000361099.8:c.*942A>G	rs41363648	A	A	hsa-miR-2113	2	gcCACAAAAttgg	D	0.003
					hsa-miR-4474-3p	2	GCCACAAaattgg	D	-0.137
					hsa-miR-5010-3p	2	gcCACAAAAttgg	D	0.01
					hsa-miR-7108-5p	2	GCCACAAaattgg	D	-0.143
				G	hsa-miR-134-3p	2	gCCACAGAattgg	C	-0.113
					hsa-miR-4778-5p	2	gccACAGAATtgg	C	0.011
					hsa-miR-7114-5p	2	gCCACAGAattgg	C	-0.104
2:190969848	ENST00000361099.8:c.*855G>A	rs188557905	G						
				A	hsa-miR-4699-3p	3	gatGTAAATAaac	C	0.043
2:190969926	ENST00000361099.8:c.*777A>G	rs180904823	A	A	hsa-miR-297	2	CATACAAttattt	D	-0.073
					hsa-miR-3149	2	CATACAAttattt	D	-0.052
					hsa-miR-5692a	3	catacaATTATTT	D	0.078
					hsa-miR-675-3p	2	CATACAAttattt	D	-0.047
2:190970033	ENST00000361099.8:c.*670T>G	rs186033487	T						
				G	hsa-miR-122-3p	2	tATGGCGTAatga	C	-0.462
2:190970106	ENST00000361099.8:c.*597C>T	rs79073086	C	C	hsa-miR-8066	2	cttTCACATTggc	D	-0.09
				G	hsa-miR-4766-5p	2	CTTTCAGAttggc	C	-0.176
2:190970129	ENST00000361099.8:c.*574T>C	rs190508584	T	T	hsa-miR-1250-3p	1	gGAAAATGtgtat	N	0.037
					hsa-miR-4643	9	ggaaaATGTGTAt	D	-0.036
					hsa-miR-466	9	ggaaaATGTGTAt	D	-0.105
					hsa-miR-4789-3p	9	ggaaaATGTGTAt	D	-0.034
2:190970130	ENST00000361099.8:c.*573A>G	rs114360225	A	A	hsa-miR-1250-3p	1	ggGAAAATGtgta	N	0.037
					hsa-miR-4446-5p	3	GGGAAAAtgtgta	D	-0.049
					hsa-miR-4643	22	gggaaaATGTGTA	D	-0.036
					hsa-miR-466	22	gggaaaATGTGTA	D	-0.105
					hsa-miR-4789-3p	22	gggaaaATGTGTA	D	-0.034

				G	hsa-miR-216b-3p	3	gggaAAGTGTGta	C	-0.119
					hsa-miR-329-3p	9	gggaaaGTGTGTA	C	-0.087
					hsa-miR-362-3p	9	gggaaaGTGTGTA	C	-0.084
					hsa-miR-3941	9	gggaaaGTGTGTA	C	-0.132
					hsa-miR-603	14	gggaaAGTGTGTA	C	-0.254
2:190970168	ENST00000361099.8:c.*535C>T	rs41476445	C	C	hsa-miR-488-3p	2	agtcctCTTTCAA	D	0.012
					hsa-miR-583	2	agtCCTCTTTcaa	D	-0.092
2:190970242	ENST00000361099.8:c.*461G>T	rs200344731	G	G	hsa-miR-935	1	ttaagtGTAACTG	N	-0.123
2:190970275	ENST00000361099.8:c.*428G>C	rs139958571	G	G	hsa-miR-150-5p	3	tctgTGGGAGAat	D	-0.145
					hsa-miR-188-3p	2	tcTGTGGGAgaat	D	-0.181
					hsa-miR-2116-3p	3	tctgTGGGAGAat	D	-0.163
					hsa-miR-4713-5p	3	tctgTGGGAGAAt	D	-0.32
					hsa-miR-532-3p	2	tctGTGGGAGAat	D	-0.396
					hsa-miR-629-3p	5	tctgtGGGAGAAt	D	-0.195
					hsa-miR-6867-3p	5	tctgtGGGAGAAt	D	-0.147
				C	hsa-miR-6814-3p	3	tctgTGCGAGAat	C	-0.249
					hsa-miR-6872-5p	3	tctgTGCGAGAat	C	-0.249
2:190970297	ENST00000361099.8:c.*406G>A	rs182394503	G	G	hsa-miR-155-3p	6	cttgacGTAGGAA	D	-0.121
					hsa-miR-3685	6	cttgacGTAGGAA	D	-0.053
					hsa-miR-598-3p	1	ctTGACGTAggaa	N	-0.151
					hsa-miR-6761-3p	1	cttgaCGTAGGAa	N	-0.209
				A	hsa-miR-202-5p	1	cttgaCATAGGAA	C	-0.251
					hsa-miR-337-3p	6	cttgacATAGGAA	C	-0.063
2:190970339	ENST00000361099.8:c.*364G>C	rs186032149	G	G	hsa-miR-4714-3p	3	gttaTAGGTTGtt	D	-0.202
2:190970399	ENST00000361099.8:c.*304A>T	rs190542524	A	A	hsa-miR-4279	1	tgggaaAGGAGAA	N	-0.082
				T	hsa-miR-136-5p	1	tgggAATGGAGAa	C	-0.196
					hsa-miR-515-5p	1	tgggaaTGGAGAA	C	-0.101
					hsa-miR-519e-5p	1	tgggaaTGGAGAA	C	-0.091
2:190970440	ENST00000361099.8:c.*263A>G	rs182725919	A	A	hsa-miR-1298-3p	7	CCAGATAcaccca	D	-0.111
					hsa-miR-3126-3p	7	CCAGATAcaccca	D	-0.126
					hsa-miR-4455	22	ccagatACACCCA	D	-0.181
					hsa-miR-609	22	ccagatACACCCA	D	-0.176
					hsa-miR-6772-5p	10	ccagaTACACCCA	D	-0.449
				G	hsa-miR-6741-5p	22	ccagatGCACCCA	C	-0.314
2:190970549	ENST00000361099.8:c.*154T>C	rs41481847	T	T	hsa-miR-1303	5	ttttTCTCTAAct	D	-0.206
					hsa-miR-3123	5	tttTTCTCTAAct	D	-0.079
					hsa-miR-3925-5p	5	tttTTCTCTAAct	D	-0.086
				C	hsa-miR-3148	3	TTTTTCCctaact	C	-0.08
					hsa-miR-3162-5p	5	ttttTCCCTAAct	C	-0.155

					hsa-miR-4668-5p	2	tTTTTCCCtaact	C	-0.155
					hsa-miR-5584-5p	4	ttTTTCCCTAact	C	-0.308
					hsa-miR-6750-5p	5	tttTTCCCTAact	C	-0.144
					hsa-miR-6822-5p	5	tttTTCCCTAact	C	-0.144

Table S2: miRNA target genes enrichment analysis by ShinyGo

Description	Number of genes	Number of genes in background	Preferred Names	p_value	FDR
Pathways in cancer	92	517	ADCY5, ADCY6, AR, ARHGEF12, ARNT, AXIN2, BCL2, CASP9, CBL, CCND1, CCND2, CCNE2, CDK6, CDKN1A, CDKN1B, CRK, CTBP2, CXCL12, DAPK2, E2F1, E2F3, EGLN2, ELK1, EML4, ERBB2, ESR2, ETS1, F2RL3, FASLG, FGF2, FGF9, FOS, FZD2, FZD5, GNAI1, GNAS, GNG12, GNG3, GRB2, GSTM5, GSTO2, HHIP, HMOX1, IFNA6, IFNAR2, IGF1, IGF1R, IKBKG, IL2RA, IL5RA, IL6, IL6R, IL6ST, ITGA3, LAMC1, LPAR3, MAPK1, MAPK3, MAX, MSH3, MYC, NCOA3, NOTCH1, NOTCH2, PDGFRA, PIK3R1, PRKCB, PTCH1, PTEN, PTGER3, RARA, ROCK1, ROCK2, RPS6KA5, SMAD2, SMAD4, SP1, STAT5A, STK4, TCEB1, TGFBR1, TGFBR2, TRAF2, TRAF5, VEGFA, VEGFB, VEGFC, WNT16, WNT2B, WNT7A, WNT9A, ZBTB16	2.04E-05	0.0069
Cellular senescence	36	150	ATM, CACNA1D, CCND1, CCND2, CCNE2, CDK6, CDKN1A, CHEK1, E2F1, E2F3, ETS1, IL6, ITPR2, LIN52, LIN9, MAPK1, MAPK14, MAPK3, MCU, MYC, NFATC2, PIK3R1, PPP1CB, PPP1CC, PPP3R1, PTEN, RAD50, RBBP4, SLC25A6, SMAD2, TGFBR1, TGFBR2, TRAF3IP2, TSC1, ZFP36L1, ZFP36L2	5.84E-05	0.0098
Hippo signaling pathway	36	153	AXIN2, BMPR1A, CCND1, CCND2, CRB2, CSNK1D, DLG4, FZD2, FZD5, GDF6, INADL, LATS2, LIMD1, MYC, NF2, NKD1, PARD3, PARD6B, PPP1CB, PPP1CC, PPP2CA, PPP2R2A, RASSF6, SAV1, SMAD2, SMAD4, SNAI2, TGFBR1, TGFBR2, TP73, WNT2B, WNT7A, WNT9A, WNT16, WTIP, YWHAZ	8.23E-05	0.0098
Human cytomegalovirus infection	45	218	ADCY5, ADCY6, AKAP13, ARHGEF12, CASP9, CCND1, CCR5, CDK6, CDKN1A, CREB1, CREB5,, CRK, CXCL12, E2F1, E2F3, ELK1, FASLG, GNAI1, GNAS, GNG12, GNG3, GRB2, IFNA6, IKBKG, IL1B, IL6, IL6R, ITPR2, MAPK1, MAPK14, MAPK3, MYC, NFATC2, PDGFRA, PIK3R1, PPP3R1, PRKCB, PTGER3, ROCK1, ROCK2, SP1, TRAF2, TRAF5, TSC1, VEGFA	0.00017	0.0098
Herpes simplex virus 1 infection	83	479	ALYREF, BCL2, C3, CASP9, FASLG, HLA-DRB5, IFNA6, IFNAR2, IKBKG, IL1B, IL6, MAVS, PIK3R1, PILRB, POU2F1, PPP1CB, PPP1CC, SRSF1, SRSF4, SRSF7, SYK, TAB2, TNFSF14, TRAF2, TRAF5, TSC1, ZFP14, ZFP30, ZFP69B, ZNF107, ZNF12, ZNF132, ZNF175, ZNF229, ZNF248, ZNF254, ZNF257, ZNF284, ZNF3, ZNF347, ZNF350, ZNF354B, ZNF398, ZNF415, ZNF426, ZNF429, ZNF431, ZNF44, ZNF440, ZNF460, ZNF468, ZNF510, ZNF543, ZNF544, ZNF548, ZNF551, ZNF556, ZNF557, ZNF562, ZNF563, ZNF568, ZNF584, ZNF610, ZNF614, ZNF616, ZNF621, ZNF665, ZNF669, ZNF670, ZNF675, ZNF680, ZNF689, ZNF705A, ZNF713, ZNF730, ZNF746, ZNF772, ZNF785, ZNF791, ZNF83, ZNF878, ZNF90, ZNF99	0.00011	0.0098
MicroRNAs in cancer	37	160	ATM, BCL2, CCND1, CCND2, CCNE2, CD44, CDK6, CDKN1A, CDKN1B, CRK, E2F1, E2F3, EFNA1, ERBB2<br>EZR, GRB2, HMOX1, IRS1, MAPK1, MAPK3, MDM4, MYC, NOTCH1, NOTCH2, PAK4, PDGFRA, PIK3R1, PRKCB, PTEN, RDX, ROCK1, RPS6KA5, STMN1, TNR<br>TRIM71, VEGFA, VIM	8.97E-05	0.0098
Proteoglycans in cancer	41	196	ANK3, ARHGEF12, CBL, CCND1, CD44, CDKN1A, ELK1<br>ERBB2, EZR, FASLG, FGF2, FZD2, FZD5, GRB2, IGF1, IGF1R, ITPR2, MAPK1, MAPK14, MAPK3, MYC, PDPK1<br>PIK3R1, PPP1CB, PPP1CC, PRKCB, PTCH1, RDX, ROCK1, ROCK2, SDC1, SMAD2, TIAM1, TLR4, TWIST1, VAV3,VEGFA, WNT16, WNT2B, WNT7A,WNT9A	0.00025	0.0121

Endocrine resistance	24	95	ADCY5, ADCY6, BCL2, CARM1, CCND1, CDKN1A, CDKN1B, E2F1, E2F3, ERBB2, ESR2, FOS, GNAS, GRB2 IGF1, IGF1R, MAPK1, MAPK14, MAPK3, NCOA3, NOTCH1, NOTCH2, PIK3R1, SP1	0.0005	0.0212
Cushing syndrome	33	153	ADCY5, ADCY6, ARNT, AXIN2, CACNA1D, CCND1 CCNE2, CDK6, CDKN1A, CDKN1B, CREB1, CREB5, E2F1, E2F3, FZD2, FZD5, GNAI1, GNAS, ITPR2, KCNA4 KMT2A, KMT2D, MAPK1, MAPK3, MC2R, NCEH1, ORAI1, SP1, STAR, WNT16, WNT2B, WNT7A, WNT9A	0.00061	0.0228
HIF-1 signaling pathway	24	106	ALDOA, ARNT, BCL2, CDKN1A, CDKN1B, CYBB, EGLN2, ERBB2, HMOX1, IGF1, IGF1R, IL6, IL6R, MAPK1 MAPK3, MKNK2, PDK1, PFKM, PIK3R1, PRKCB, TCEB1 TFRC, TLR4, VEGFA	0.0018	0.0361
FoxO signaling pathway	28	127	ATG12, ATM, CCND1, CCND2, CDKN1A, CDKN1B, FASLG, G6PC, GABARAP, GABARAPL1, GRB2, IGF1, IGF1R, IL6, IRS1, MAPK1, MAPK14, MAPK3, PDPK1, PIK3R1, PRKAA2, PRKAB2, PTEN, SMAD4, SOD2, STK4, TGFBR1, TGFBR2	0.0011	0.0361
p53 signaling pathway	19	72	AIFM2, ATM, BCL2, CASP9, CCND1, CCND2, CCNE2, CD82, CDK6, CDKN1A, CHEK1, IGF1, MDM4, PERP, PTEN, SESN3, SFN, TP73, ZMAT3	0.0012	0.0361
Axon guidance	35	177	ABLIM1, ARHGEF12, CFL1, CFL2, CXCL12, EFNA1, EPHB3, EPHB4, GNAI1, LRRC4, MAPK1, MAPK3, NFATC2, PAK2, PAK4, PARD3, PARD6B, PDK1, PIK3R1 PLXNC1, PPP3R1, PTCH1, ROCK1, ROCK2, SEMA3E SEMA3G, SEMA4C, SEMA4F, SEMA4G, SLIT1, SRGAP1 SRGAP2, TRPC5, UNC5B, UNC5C	0.0016	0.0361
Oxytocin signaling pathway	31	149	ADCY5, ADCY6, CACNA1D, CACNA2D2, CACNG8, CAMKK2, CCND1, CDKN1A, EEF2K, ELK1, FOS, GNAI1 GNAS, ITPR2, KCNJ12, KCNJ3, KCNJ6, KCNJ9, MAPK1 MAPK3, MYLK3, NFATC2, PIK3CG, PPP1CB, PPP1CC PPP3R1, PRKAA2, PRKAB2, PRKCB, ROCK1, ROCK2	0.0014	0.0361
Hepatitis B	33	159	BCL2, CASP9, CCNE2, CDKN1A, CREB1, CREB5, DDB1 DDX3X, E2F1, E2F3, EGR3, ELK1, FASLG, FOS, GRB2, IFNA6, IKBKG, IL6, MAPK1, MAPK14, MAPK3, MAVS, MYC, NFATC2, PIK3R1, PRKCB, SMAD4, STAT5A, TAB2, TGFBR, TGFBR2, TLR4, YWHAZ	0.0011	0.0361
Human T-cell leukemia virus 1 infection	40	211	ADCY5, ADCY6, ATM, BUB3, CCND1, CCND2, CCNE2 CDC23, CDKN1A, CHEK1, CREB1, CREB5, E2F1, E2F3 ELK1, ELK4, ETS1, FOS, HLA-DRB5, ICAM1, IKBKG, IL2RA, IL6, MAPK1, MAPK3, MYC, NFATC2, PIK3R1 PPP3R1, PTEN, SLC25A6, SMAD2, SMAD4, STAT5A TBPL1, TBPL2, TGFBR1, TGFBR2, TNFRSF13C, VAC14	0.0015	0.0361
Prostate cancer	23	96	AR, BCL2, CASP9, CCND1, CCNE2, CDKN1A, CDKN1B CREB1, CREB5, E2F1, E2F3, ERBB2, ERG, GRB2, IGF1 IGF1R, IKBKG, MAPK1, MAPK3, PDGFRA, PDPK1, PIK3R1, PTEN	0.0012	0.0361
Chronic myeloid leukemia	19	75	CBL, CCND1, CDK6, CDKN1A, CDKN1B, CRK, CTBP2, E2F1, E2F3, GRB2, IKBKG, MAPK1, MAPK3, MYC, PIK3R1, SMAD4, STAT5A, TGFBR1, TGFBR2	0.0018	0.0361
MAPK signaling pathway	50	288	CACNA1A, CACNA1D, CACNA2D2, CACNG8, CRK, DUSP10, DUSP4, DUSP6, EFNA1, ELK1, ELK4, ERBB2, EREG, FASLG, FGF2, FGF9, FOS, GNG12, GRB2, HSPA6 HSPA8, IGF1, IGF1R, IKBKG, IL1B, LAMTOR3, MAP3K2 MAPK1, MAPK14, MAPK3, MAPK8IP3, MAX, MKNK2 MYC, PAK2, PDGFRA, PPM1A, PPP3R1, PRKCB, RPS6KA5, STK4, STMN1, TAB2, TAOK1, TGFBR1, TGFBR2, TRAF2, VEGFA, VEGFB, VEGFC	0.0023	0.0406
Viral carcinogenesis	35	182	C3, CCND1, CCND2, CCNE2, CCR5, CDK6, CDKN1A, CDKN1B, CHEK1, CREB1, CREB5, DDB1, DDX3X, EGR3, GRB2, GSN, GTF2H3, IKBKG, IL6ST, LYN, MAPK1, MAPK3, PIK3R1,PKM, REL, SCIN, SND1, STAT5A, SYK, TBPL1, TBPL2, TRAF2, TRAF5, VAC14, YWHAZ	0.0024	0.0406
Transcriptional misregulation in cancer	33	171	AFF1, ATM, BMP2K, CCND2, CCNT1, CDK9, CDKN1A, CDKN1B, DUSP6, ELK4, ERG, HOXA10, HOXA11, IGF1, IGF1R, IL6, KMT2A, LDB1, MAX, MYC,MYCN, PAX3, RARA, REL, SIN3A, SIX1, SIX4, SP1, SS18, TGFBR2, TLX1, WNT16, ZBTB16	0.003	0.0476

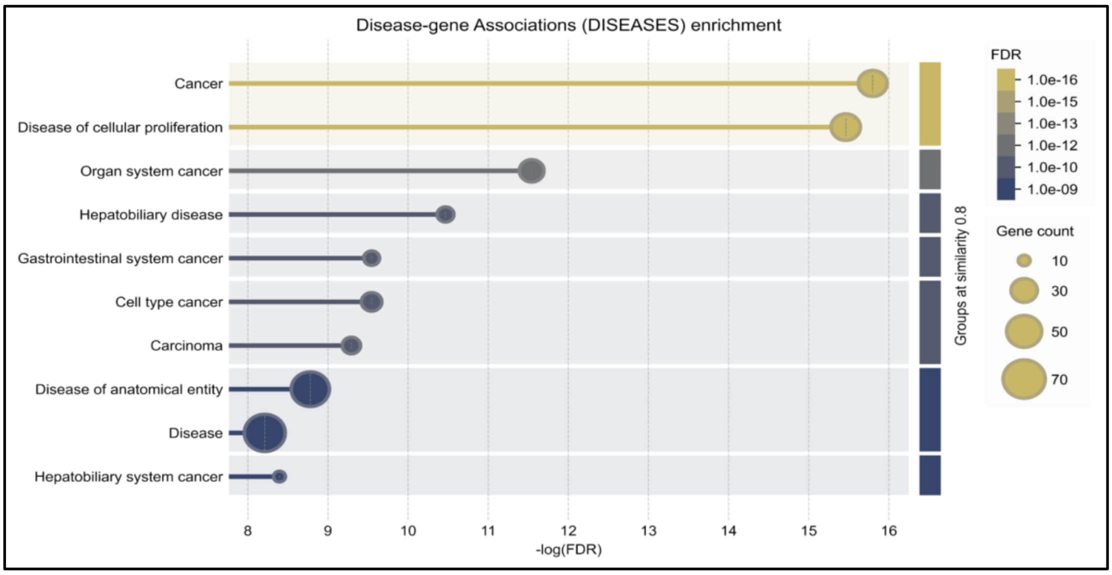

Figure S1. Disease-gene association enrichments miRNAs' target genes using STRING database.

This figure illustrates the disease- gene associations. The y-axis lists different diseases, while the x-axis represents the negative logarithm of the False Discovery Rate (FDR), indicating the significance of enrichment. Each circle corresponds to a specific disease, with the size of the circle representing the gene count associated with that disease. The light yellow to dark blue colour gradient indicates the range of FDR values, where a lower FDR suggests higher statistical significance

(A)

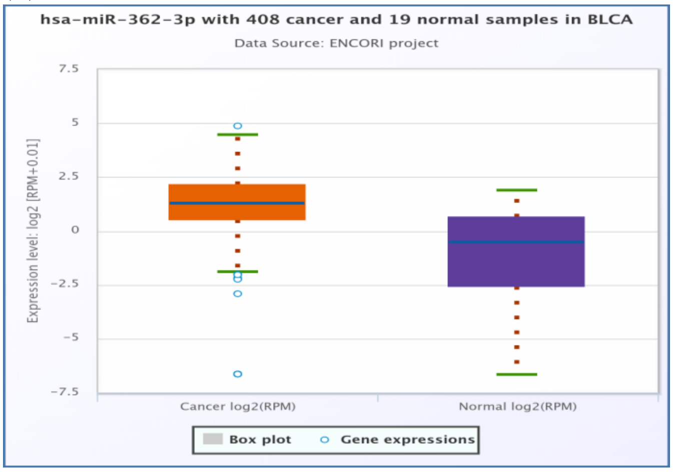

P value 2.4e-9

(B)

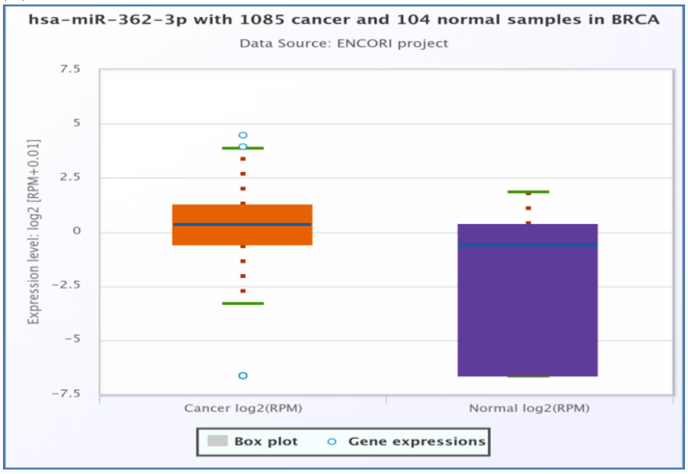

P value 1.9e-8

(C)

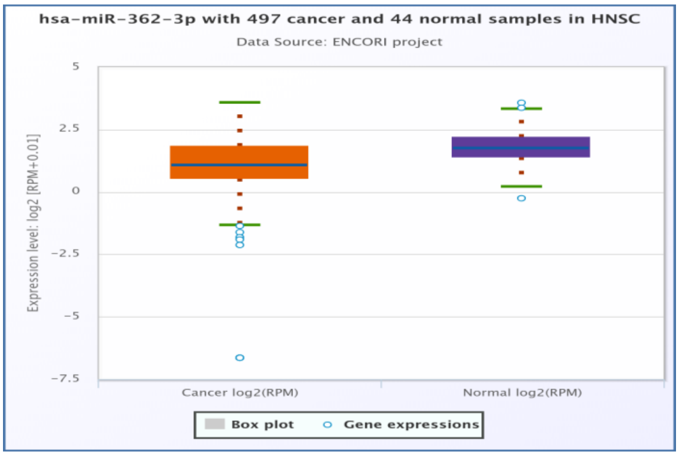

P value 4.3e-5

(D)

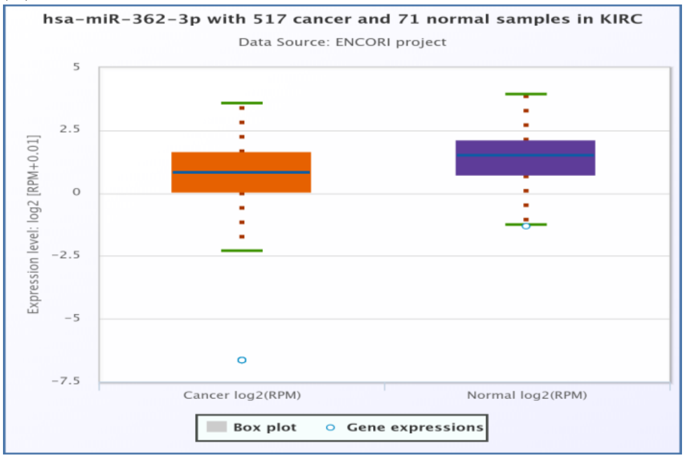

P value 0.00017

(E)

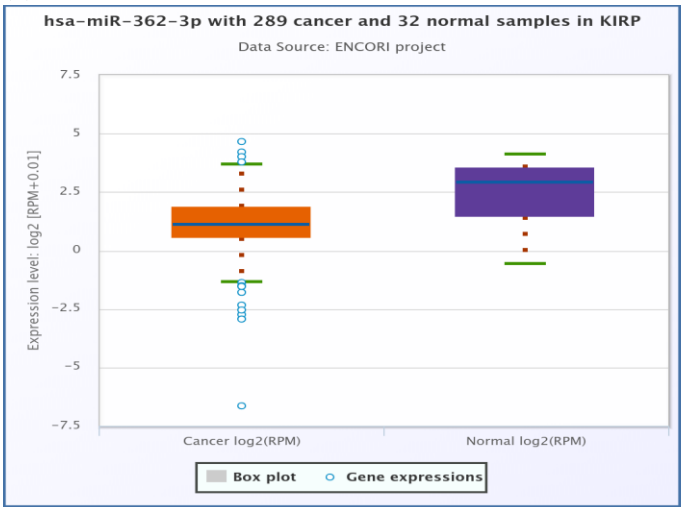

P value 5.4e-10

(F)

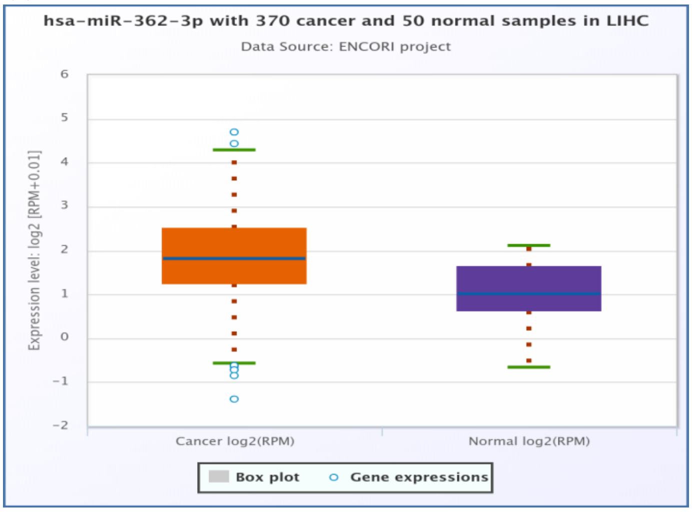

P value 8.7e-9

(G)

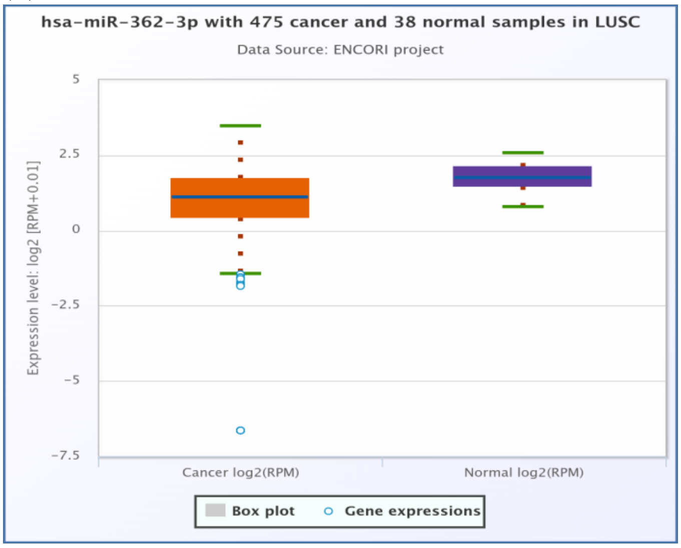

P value 0.00037

(H)

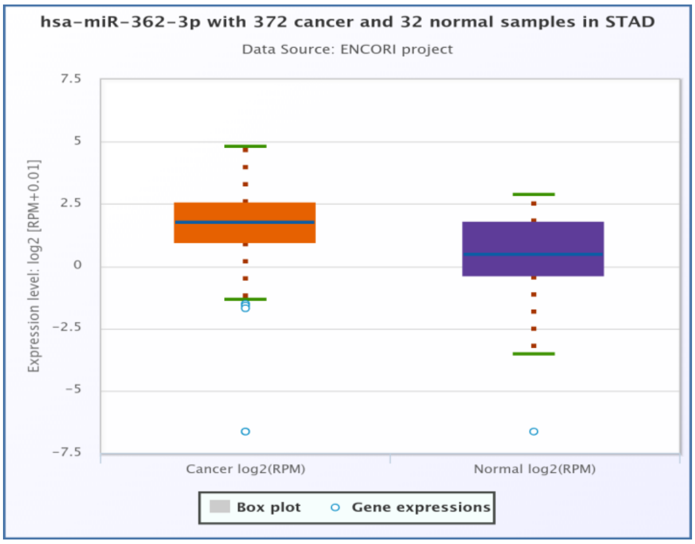

P value 2.1e-7

(I)

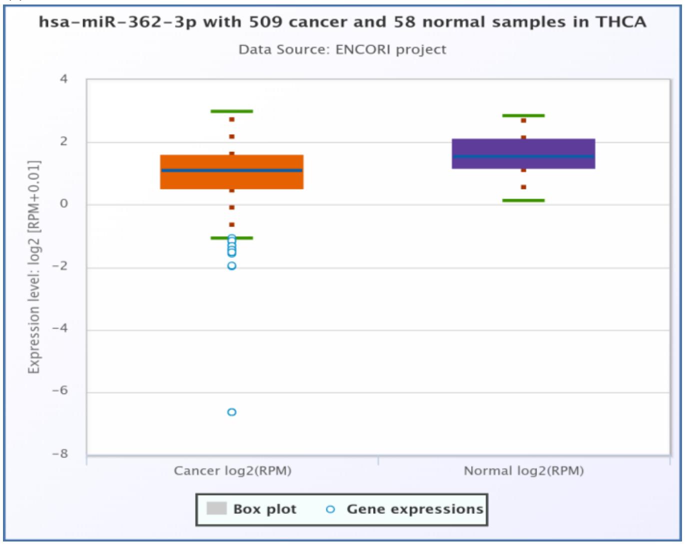

P value 2.3e-5

(J)

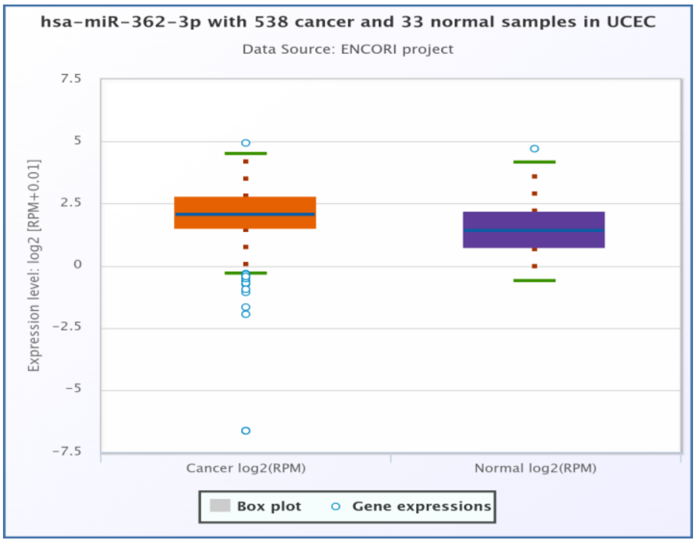

P value 0.034

(K)

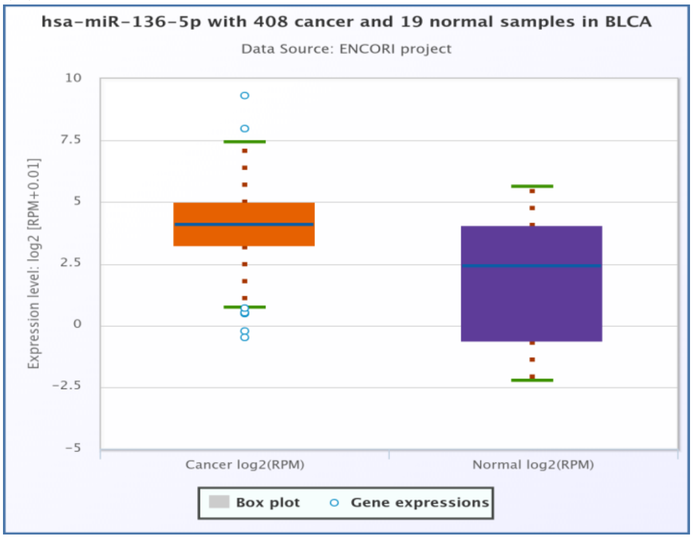

P value 4.9e-10

(L)

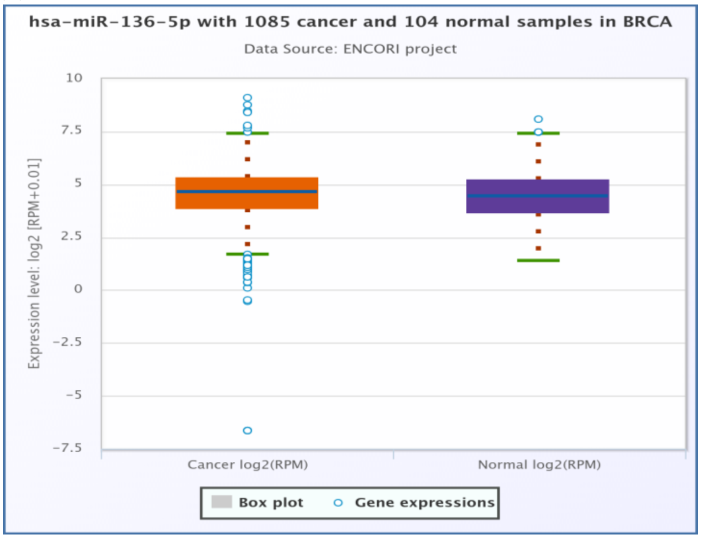

P value 0.82

(M)

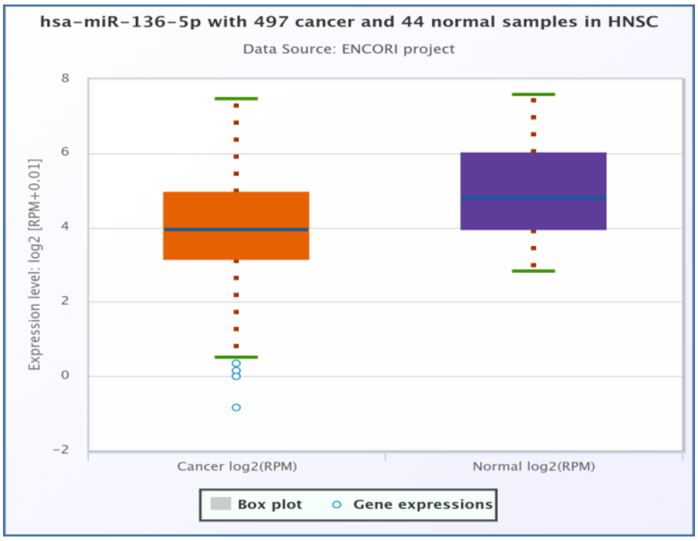

P value 1.4e-6

(N)

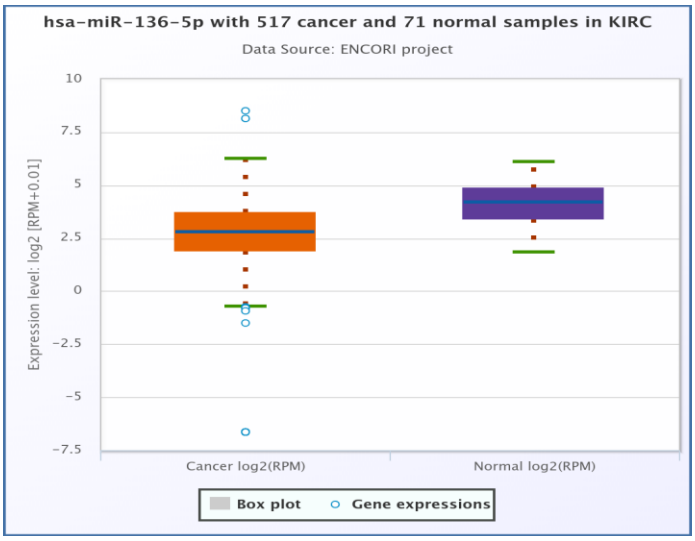

P value 1.2e-13

(O)

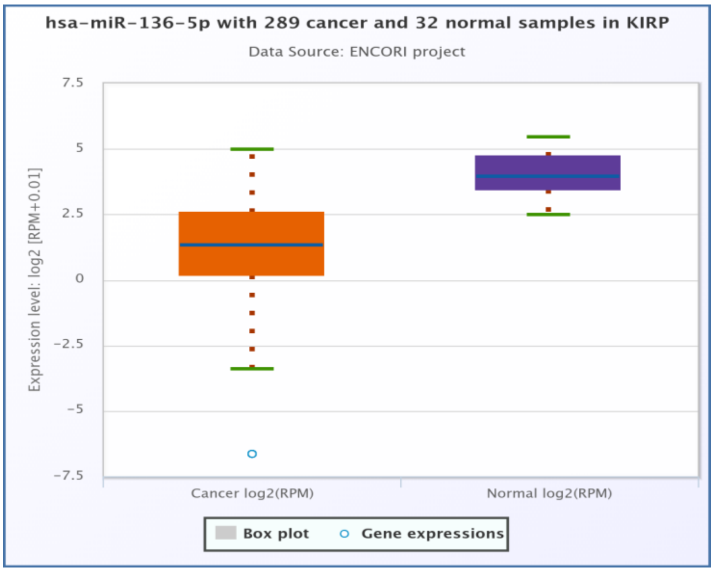

P value 3.0e-13

(P)

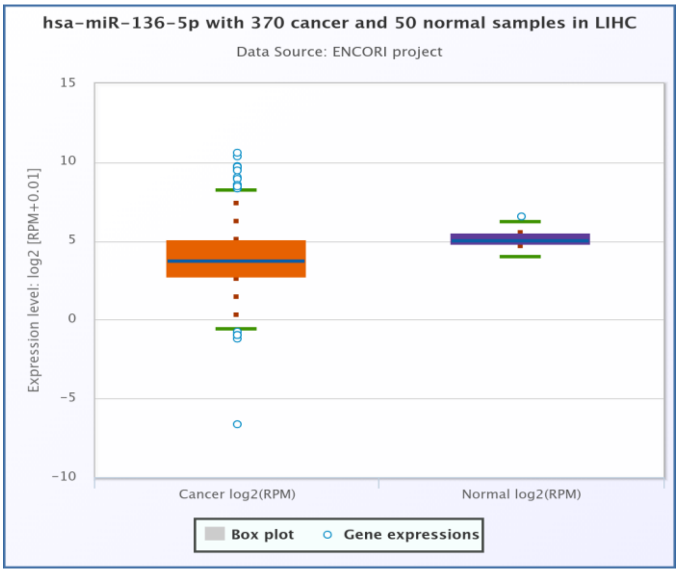

P value 2.1e-5

(Q)

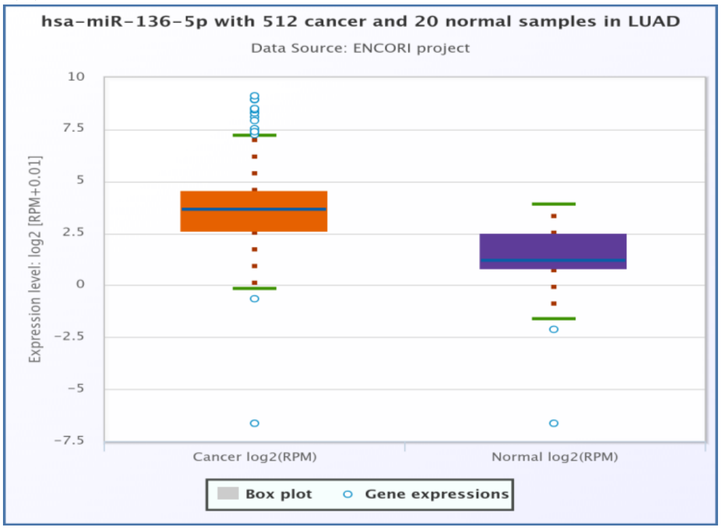

P value 1.7e-11

(R)

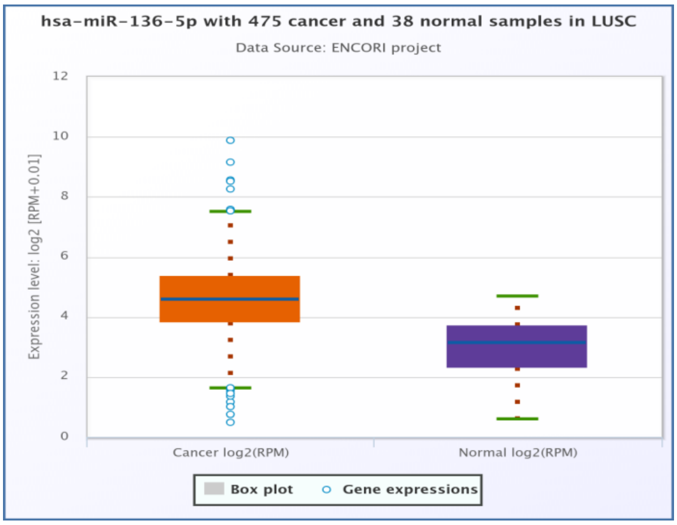

P value 6.3e-15

(S)

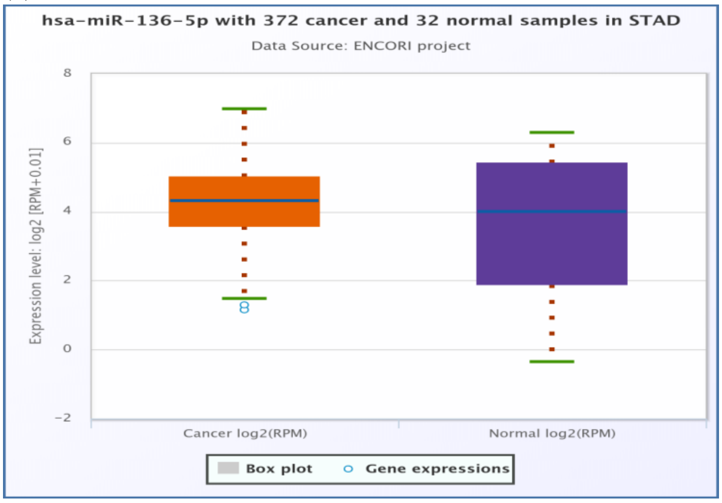

P value 0.0024

(T)

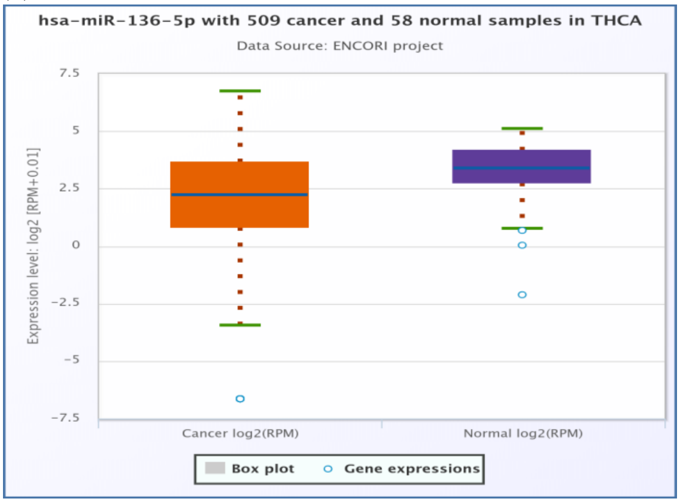

P value 3.0e-5

(U)

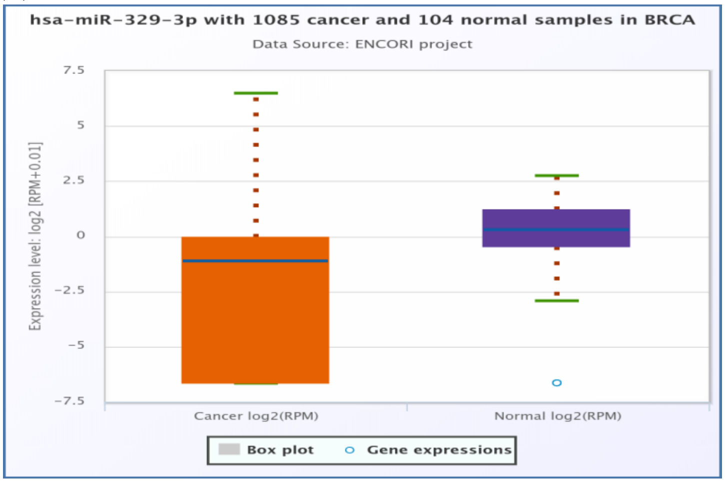

P value 3.4e-15

(V)

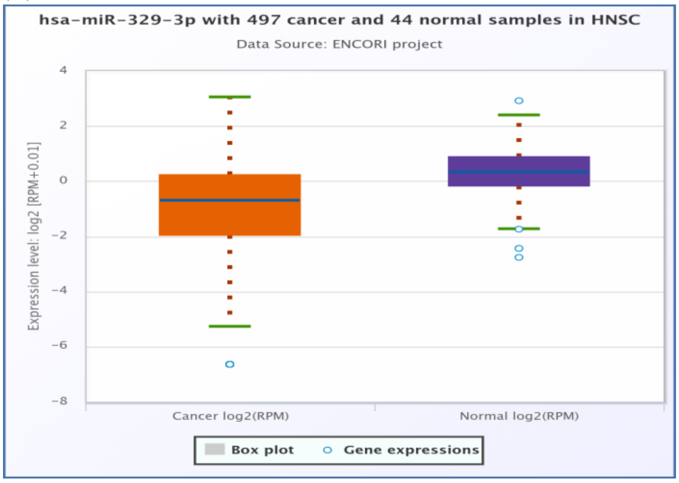

P value 1.3e-5

(W)

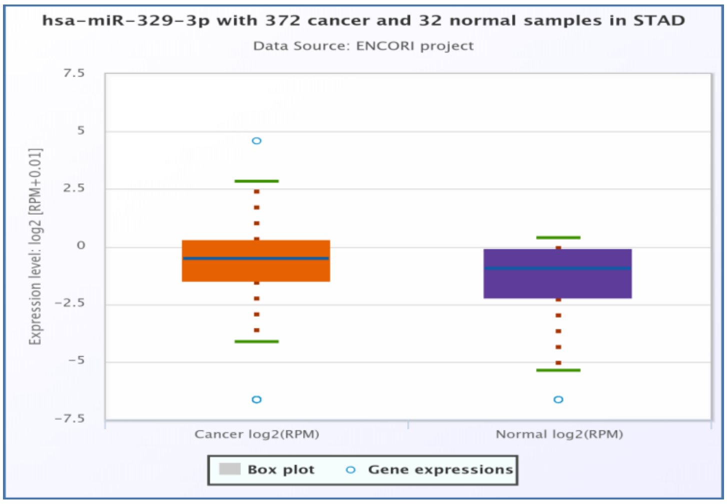

P value 0.23

(X)

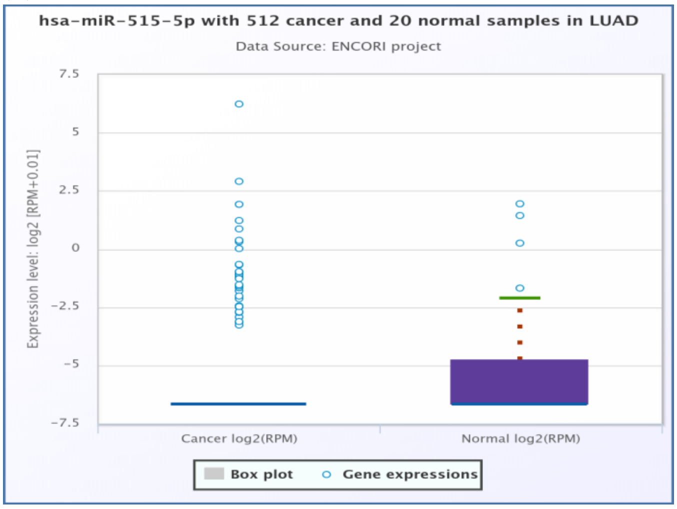

P value 0.0003

Figure S2. The expression analysis of the four significant differentially expressed miRNAs (hsa-miR-362-3p (A-J), hsa-miR-136-5p (K-T), hsa-miR-329-3p (T-W)) and hsa-miR-515-5p (X), in human cancer using StarBase database.

P-value<0.05 is considered significant.

(A)

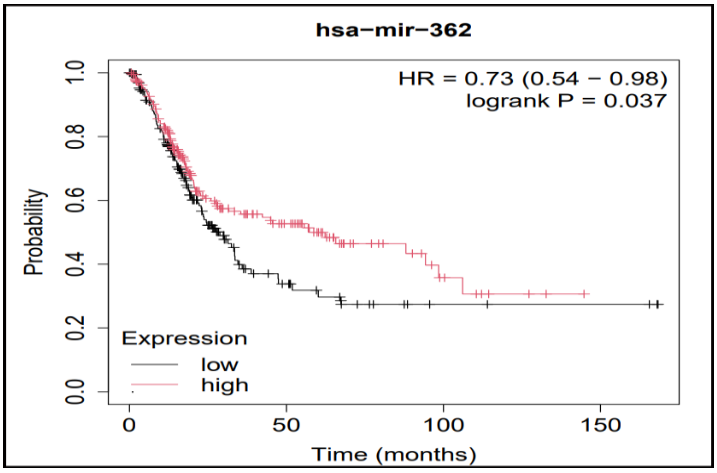

BLCA

(B)

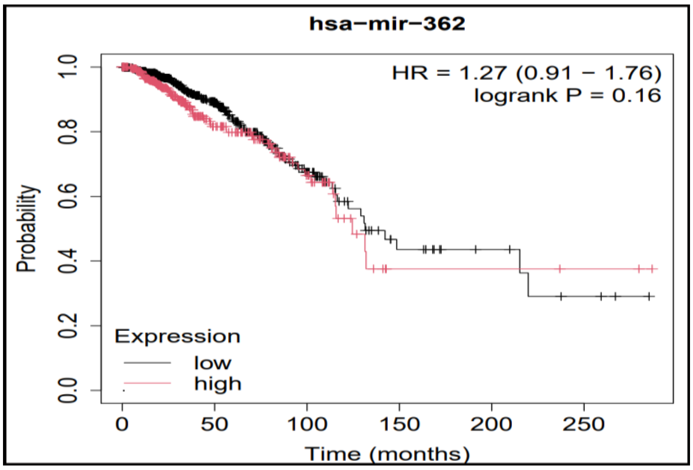

BRCA

(C)

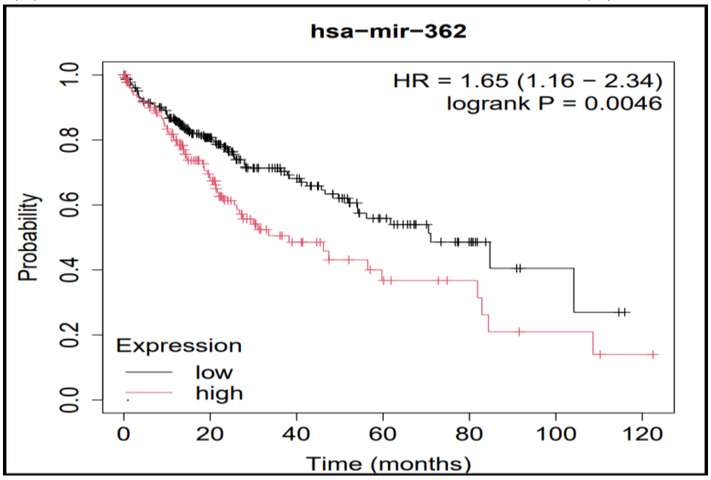

LIHC

(D)

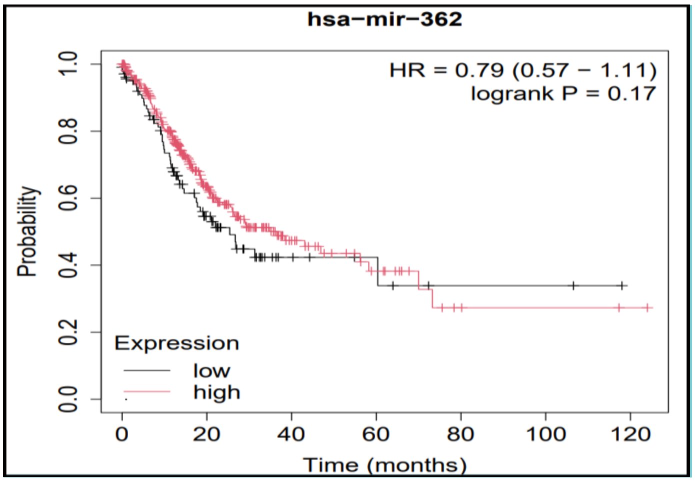

STAD

(E)

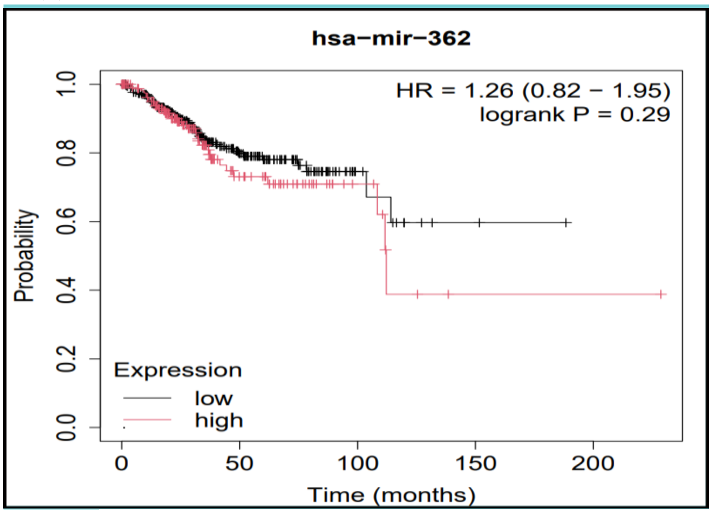

UCEC

(F)

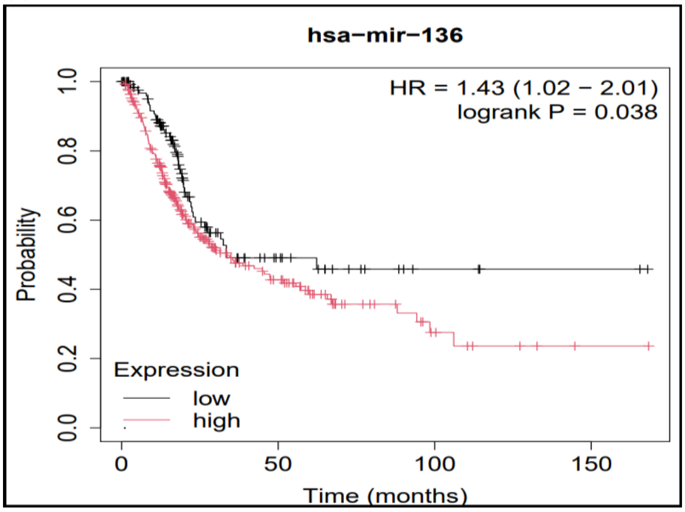

BLCA

(G)

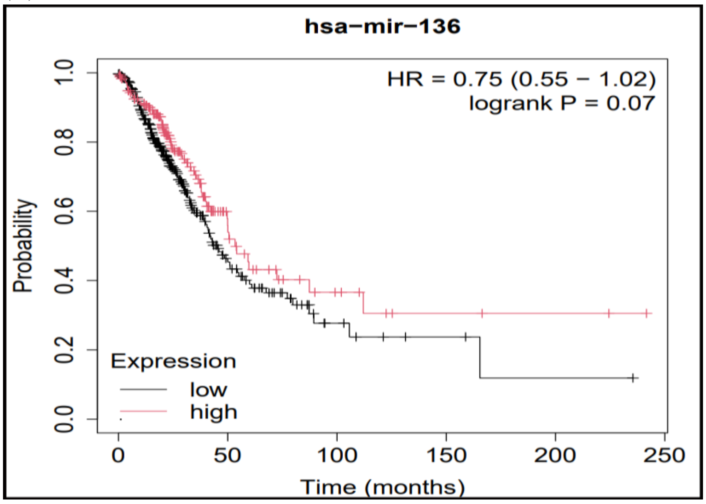

LUAD

(H)

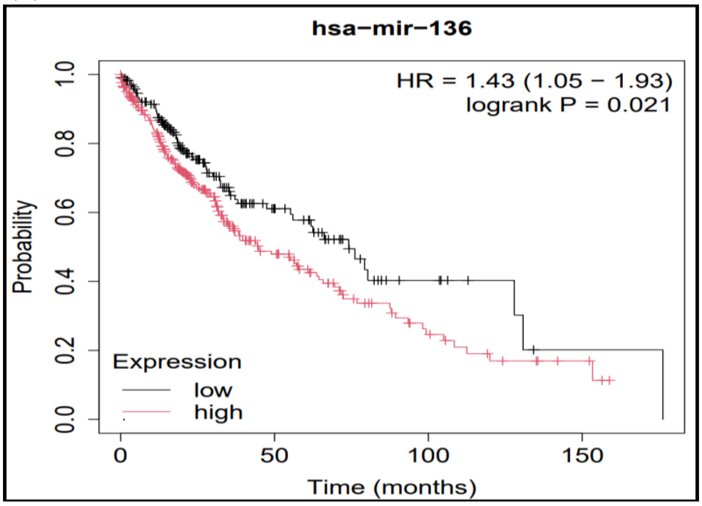

LUSC

(I)

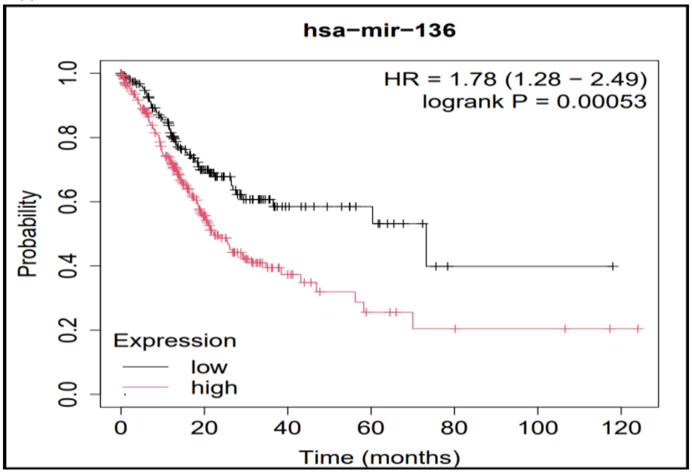

STAD

Figure S3. Survival analysis of the significantly up-regulated miRNAs in cancer (hsa-miR-362(A-D), hsa-miR-136(E-I), using Kaplan Meier Plotter.
